# The Realizability of Theta Graphs as Reconfiguration Graphs of Minimum Independent Dominating Sets

**DOI:** 10.2478/amsil-2024-0002

**Published:** 2024-02-21

**Authors:** R.C. Brewster, C.M. Mynhardt, L.E. Teshima

**Affiliations:** Department of Mathematics and Statistics, Thompson Rivers University, 805 TRU Way, Kamloops, B.C., Canada V2C 0C8; Department of Mathematics and Statistics, University of Victoria, PO BOX 1700 STN CSC, Victoria, B.C., Canada V8W 2Y2

**Keywords:** 05C69, independent domination number, graph reconfiguration, *i*-graph, theta graph

## Abstract

The independent domination number *i*(*G*) of a graph *G* is the minimum cardinality of a maximal independent set of *G*, also called an *i*(*G*)-set. The *i*-graph of *G*, denoted *ℐ* (*G*), is the graph whose vertices correspond to the *i*(*G*)-sets, and where two *i*(*G*)-sets are adjacent if and only if they differ by two adjacent vertices. Not all graphs are *i*-graph realizable, that is, given a target graph *H*, there does not necessarily exist a source graph *G* such that *H* ≅ *ℐ* (*G*). We consider a class of graphs called “theta graphs”: a theta graph is the union of three internally disjoint nontrivial paths with the same two distinct end vertices. We characterize theta graphs that are *i*-graph realizable, showing that there are only finitely many that are not. We also characterize those line graphs and claw-free graphs that are *i*-graphs, and show that all 3-connected cubic bipartite planar graphs are *i*-graphs.

## 1. Introduction

Our main topic is the characterization of theta graphs that are obtainable as reconfiguration graphs with respect to the minimum independent dominating sets of some graph.

In graph theory, reconfiguration problems are often concerned with solutions to a specific problem that are vertex subsets of a graph. When this is the case, the reconfiguration problem can be viewed as a token manipulation problem, where a solution subset is represented by placing a token at each vertex of the subset. Each solution is represented as a vertex of a new graph, referred to as a *reconfiguration graph*, where adjacency between vertices corresponds to a predefined token manipulation rule called the *reconfiguration step*. The reconfiguration step we consider here consists of sliding a single token along an edge between adjacent vertices belonging to different solutions.

More formally, given a graph *G*, the *slide graph* of *G* is the graph *H* such that each vertex of *H* represents a solution of some problem on *G*, and two vertices *u* and *v* of *H* are adjacent if and only if the solution in *G* corresponding to *u* can be transformed into the solution corresponding to *v* by sliding a single token along the edge *uv* ∈ *E*(*G*). See [7] for a survey on reconfiguration of colourings and dominating sets in graphs.

We use the standard notation of *α*(*G*) for the independence number of a graph *G*. The *independent domination number i*(*G*) of *G* is the minimum cardinality of a maximal independent set of *G*, or, equivalently, the minimum cardinality of an independent domination set of *G*. An independent dominating set of *G* of cardinality *i*(*G*) is also called an *i*-*set* of *G*, or an *i*(*G*)-*set*. An *α*-*set* of *G*, or an *α*(*G*)-*set*, is defined similarly. When *i*(*G*) = *α*(*G*), we say that *G* is *well-covered*.

Given a graph *G*, we consider the slide graph *ℐ* (*G*) of *G*, formally defined in Section 2 below, with respect to its *i*-sets. Our main result, Theorem 5.3, concerns the class Θ of “theta graphs” for which we characterize, in Sections 5 and 6, those graphs *H* ∈ Θ for which there exists a graph *G* such that *H* ≅ *ℐ* (*G*). We state known results required here in Section 3. We introduce the technique we use for theta graphs in Section 4, where we apply it to the simpler problem of characterizing line graphs and claw-free graphs that are realizable as *i*-graphs. In Section 7.1 we exhibit a graph that is neither a theta graph nor an *i*-graph, and in Section 7.2 we show that certain planar graphs are *i*-graphs and *α*-graphs. We conclude with some open problems in Section 8.

In general, we follow the notation of [3]. For other domination principles and terminology, see [4, 5].

## 2. *i*-graphs and theta graphs

The *i-graph* of a graph *G*, denoted *ℐ* (*G*) = (*V* (*ℐ* (*G*)), *E*(*ℐ* (*G*))), is the graph with vertices representing the minimum independent dominating sets of *G* (that is, the *i*-sets of *G*), and where *u, v* ∈ *V* (*ℐ* (*G*)), corresponding to the *i*(*G*)-sets *S_u_* and *S_v_*, respectively, are adjacent in *ℐ* (*G*) if and only if there exists *xy* ∈ *E*(*G*) such that *S_u_* = (*S_v_ − x*) ∪ {*y*}. Imagine that there is a token on each vertex of an *i*-set *S* of *G*. Then *S* is adjacent, in *ℐ* (*G*), to an *i*(*G*)-set *S^′^* if and only if a single token can slide along an edge of *G* to transform *S* into *S^′^*. Similarly, the *α-graph 𝒜* (*G*) of a graph *G* is the slide reconfiguration graph with vertices representing the *α*(*G*)-sets, and where adjacency is defined as for the *i*-graph. In Section 5 we present several constructions for *i*-graphs that are also constructions for *α*-graphs.

We say *H is an i-graph*, or is *i*-*graph realizable*, if there exists some graph *G* such that *ℐ* (*G*) ≅ *H*. Moreover, we refer to *G* as the *seed graph* of the *i*-graph *H*. Going forward, we mildly abuse notation to denote both the *i*-set *X* of *G* and its corresponding vertex in *H* as *X*, so that *X* ⊆ *V* (*G*) and *X* ∈ *V* (*H*).

In acknowledgment of the slide-action in *i*-graphs, given *i*-sets *X* = {*x*_1_, *x*_2_, . . . , *x_k_*} and *Y* = {*y*_1_, *x*_2_, . . . *x_k_*} of *G* with *x*_1_*y*_1_ ∈ *E*(*G*), we denote the adjacency of *X* and *Y* in *ℐ* (*G*) as $X\overset{x_{1}y_{1}}{∼}Y$ , where we imagine transforming the *i*-set *X* into *Y* by sliding the token at *x*_1_ along an edge to *y*_1_. When discussing several graphs, we use the notation $X{\overset{x_{1}y_{1}}{∼}}_{G}Y$ to specify that the relationship is on *G*. More generally, we use *x* ∼ *y* to denote the adjacency of vertices *x* and *y* (and *x* ≁ *y* to denote non-adjacency); this is used in the context of both the seed graph and the target graph.

The study of *i*-graphs was initiated by Teshima in [9]. In [2], Brewster, Mynhardt and Teshima investigated *i*-graph realizability and proved some results concerning the adjacency of vertices in an *i*-graph and the structure of their associated *i*-sets in the seed graph. They presented the three smallest graphs that are not *i*-graphs: the diamond graph 𝔇 = *K*_4_ − *e*, *K*_2,3_ and the graph *κ*, which is *K*_2,3_ with an edge subdivided. They showed that several graph classes, like trees and cycles, are *i*-graphs. They demonstrated that known *i*-graphs can be used to construct new *i*-graphs and applied these results to build other classes of *i*-graphs, such as block graphs, hypercubes, forests, cacti, and unicyclic graphs.

The diamond 𝔇, *K*_2,3_, and *κ* are examples of *theta graphs*: graphs that are the union of three internally disjoint nontrivial paths with the same two distinct end vertices. The graph Θ 〈*j, k, ℓ*〉, where *j* ≤ *k* ≤ *ℓ*, is the theta graph with paths of lengths *j*, *k*, and *ℓ*. In this notation, the three non-*i*-graph realizable examples are 𝔇 ≅ Θ 〈1, 2, 2〉, *K*_2,3_ ≅ Θ 〈2, 2, 2〉, and *κ* ≅ 〈2, 2, 3〉.

## 3. Previous results

To begin, we state several useful observations and lemmas from [2, 9] about the structure of *i*-sets within given *i*-graphs. Given a set *S* ⊆ *V* (*G*) and a vertex *v* ∈ *S*, the *private neighbourhood* of *v* with respect to *S* is the set pn(*v, S*) = *N*[*v*] − *N*[*S −* {*v*}], and the *external private neighbourhood* of *v* with respect to *S* is the set epn(*v, S*) = pn(*v, S*) *−* {*v*}.

**Observation 3.1 ([2, 9]).**

*Let G be a graph and H* = *ℐ* (*G*). *A vertex X* ∈ *V* (*H*) *has* deg_*H*_(*X*) ≥ 1 *if and only if for some v* ∈ *X* ⊆ *V* (*G*), *there exists u* ∈ epn(*v, X*) *such that u dominates* pn(*v, X*).

For some path *X*_1_, *X*_2_, . . . , *X_k_* in *H*, at most one vertex of the *i*-set is changed at each step, and so *X*_1_ and *X_k_* differ on at most *k −* 1 vertices. This yields the following immediate observation.

**Observation 3.2 ([2, 9]).**

*Let G be a graph and H* = *ℐ* (*G*). *Then for any i-sets X and Y of G, the distance d_H_*(*X, Y*) ≥ |*X − Y*|.

**Lemma 3.3 ([2, 9]).**

*Let G be a graph with H* = *ℐ* (*G*). *Suppose XY and YZ are edges in H with* $X\overset{xy_{1}}{∼}Y$ *and* $X\overset{y_{2}z}{∼}Y$ , *with X* ≠ *Z. Then XZ is an edge of H if and only if y*_1_ = *y*_2_.

Combining the results from Lemma 3.3 with Observation 3.2 yields the following observation for vertices of *i*-graphs at distance 2.

**Observation 3.4 ([2, 9]).**

*Let G be a graph and H* = *ℐ* (*G*). *Then for any i-sets X and Y of G, if d_H_*(*X, Y*) = 2, *then* |*X − Y*| = 2.

**Lemma 3.5 ([2, 9]).**

*Let G be a graph and H* = *ℐ* (*G*). *Suppose H contains an induced K*_1,*m*_ *with vertex set* {*X, Y*_1_, *Y*_2_, . . . , *Y_m_*} *and* deg_*H*_(*X*) = *m. Let i* ≠ *j. Then in G,*

(i) *X − Y_i_* ≠ *X − Y_j_,*
(ii) |*Y_i_* ∩ *Y_j_*| = *i*(*G*) − 2, *and*
(iii) *m* ≤ *i*(*G*).

Lemma 3.3 and Observation 3.2 are also used to prove the next result.

**Proposition 3.6 ([2, 9]).**

**Figure 1.**
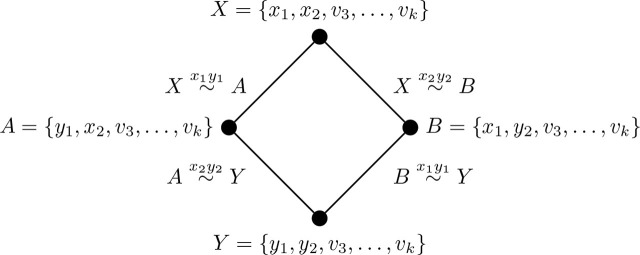
Reconfiguration structure of an induced *C*_4_ subgraph from Proposition 3.6

*Let G be a graph and H* = *ℐ* (*G*). *Suppose H has an induced C*_4_ *with vertices X, A, B, Y, where XY, AB* ∉ *E*(*H*). *Then, without loss of generality, the set composition of X, A, B, Y in G, and the edge labelling of the induced C*_4_ *in H, are as in Figure* 1.

We state the above-mentioned results regarding 𝔇, *K*_2,3_, and *κ* here for referencing.

**Proposition 3.7 ([2, 9]).**

*The graphs* 𝔇, *K*_2,3_, *and κ are not i-graph realizable.*

**Figure 2.**
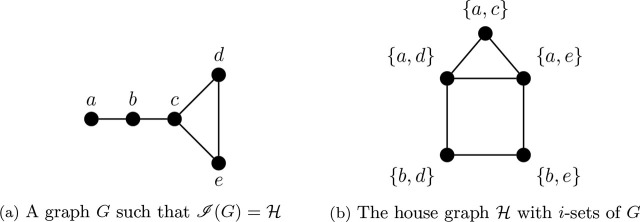
The graph *G* for Proposition 3.8 with *ℐ* (*G*) = *ℋ*

On the other hand, the house graph *ℋ* = Θ 〈1, 2, 3〉 in Figure 2(b) is a theta graph that is an *i*-graph, as illustrated by the seed graph *G* in Figure 2(a). The *i*-sets of *G* and their adjacencies are overlaid on *ℋ* in Figure 2(b).

**Proposition 3.8 ([2, 9]).**

*The house graph ℋ is an i-graph.*

The next result shows that maximal cliques in *i*-graphs can be replaced by arbitrarily larger maximal cliques to form larger *i*-graphs.

**Lemma 3.9 (Max Clique Replacement Lemma [2, 9]).**

Let H be an i-graph with a maximal m-vertex clique, 𝒦_m_. The graph H_w_ formed by adding a new vertex w* adjacent to all of 𝒦_m_ is also an i-graph.

The Deletion Lemma below shows that the class of *i*-graphs is closed under vertex deletion, that is, every induced subgraph of an *i*-graph is also an *i-*graph.

**Lemma 3.10 (Deletion Lemma [2, 9]).**

*If H is a nontrivial i-graph, then any induced subgraph of H is also an i-graph.*

**Corollary 3.11 ([2, 9]).**

*If H is not an i-graph, then any graph containing an induced copy of H is also not an i-graph.*

When visualizing the connections between the *i*-sets of a graph *G*, it is sometimes advantageous to consider its complement *G̅* instead. From a human perspective, it is curiously easier see to which vertices a vertex *v* is adjacent, rather than to which vertices *v* is nonadjacent. This is especially true when *i*(*G*) = 2 or 3, when we may interpret the adjacency of *i*-sets of *G* as the adjacencies of edges and triangles (i.e. *K*_3_), respectively, in *G̅*. In the following sections we examine how the use of graph complements can be exploited to construct the *i*-graph seeds for certain classes of line graphs, theta graphs, and maximal planar graphs.

## 4. Line graphs and claw-free graphs

Consider a graph *G* with *i*(*G*) = 2 and where *X* = {*u, v*} is an *i*-set of *G*. In *G̅*, *u* and *v* are adjacent, so *X* is represented as the edge *uv*. Moreover, no other vertex *w* is adjacent to both vertices of *X* in *G̅*; otherwise, {*u, v, w*} is independent in *G*, contrary to *X* being an *i*-set.

Now consider the line graph *L*(*G̅*) of *G̅*. If *X* = {*u, v*} is an *i*-set of *G*, then *e* = *uv* is an edge of *G̅* and hence *e* is a vertex of *L*(*G̅*). Thus, the *i*-sets of *G* correspond to a subset of the vertices of *L*(*G̅*). In the case where *G̅* is triangle-free (that is, *G* has no independent sets of cardinality 3), these *i*-sets of *G* are exactly the vertices of *L*(*G̅*). Now suppose *Y* is an *i*-set of *G* adjacent to *X*; say, $X\overset{\mathrm{uw}}{∼}Y$ , so that *Y* = {*v, w*}. Then, in *G̅*, *f* = *vw* is an edge, and so in *L*(*G̅*), *f* ∈ *V* (*L*(*G̅*)). Since *e* and *f* are both incident with *v* in *G̅*, *ef* ∈ *E*(*L*(*G̅*)). That is, for *i*-sets *X* and *Y* of a well-covered graph *G* with *i*(*G*) = *α*(*G*) = 2, *X* ∼ *Y* if and only if *X* and *Y* correspond to adjacent vertices in *L*(*G̅*). Thus *ℐ* (*G*) ≅ *L*(*G̅*).

**Figure 3.**
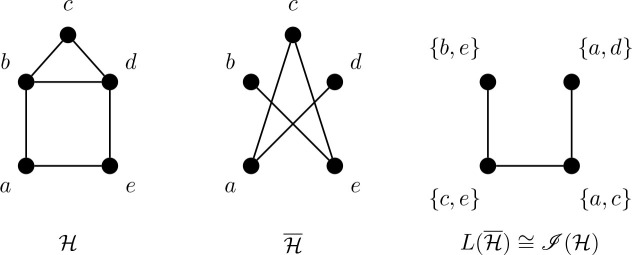
The complement and line graphs complement of the well-covered house graph *ℋ*

In the example illustrated in Figure 3 below, *ℋ* is the house graph, where *X* = {*a, c*} and *Y* = {*c, e*} are *i*-sets with $X\overset{\mathrm{ae}}{∼}Y$ . In $L\left(\bar{ℋ}\right)$ , the two vertices in each of these *i*-sets are likewise adjacent.

The connection between graphs with *i*(*G*) = 2 and line graphs helps us not only understand the structure of *ℐ* (*G*), but also lends itself towards some interesting realizability results. We follow this thread for the remainder of this section, and build towards determining the *i*-graph realizability of line graphs and claw-free graphs. The straightforward proof of the following lemma can be found in [9] and is omitted here.

**Lemma 4.1.**

**Figure 4.**
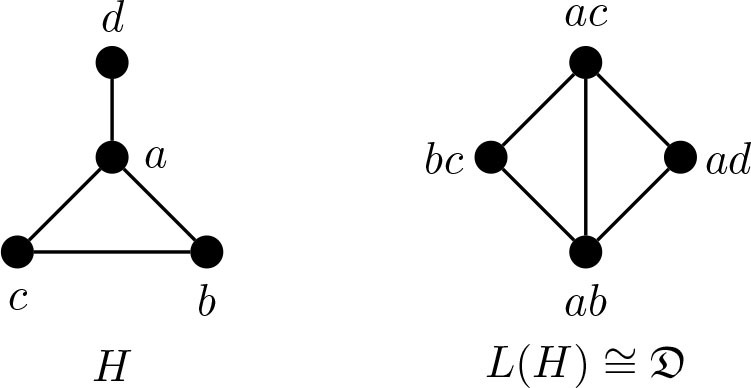
The “paw” *H* with *L*(*H*) ≅ 𝔇

*The line graph of a connected graph G of order at least* 4 *contains* 𝔇 *as an induced subgraph if and only if G contains a triangle. (See Figure* 4.*)*

**Theorem 4.2.**

*Let H be a connected line graph. Then H is an i-graph if and only if H is* 𝔇-*free.*

**Proof**

Suppose *H* is an *i*-graph. By Proposition 3.7 and Corollary 3.11, *H* is 𝔇-free.

Conversely, suppose that *H* is 𝔇-free. If *H* is complete, then *H* is the *i-*graph of itself. So, assume that *H* is not complete. Say *H* is the line graph of some graph *F*, where we may assume *F* has no isolated vertices (as isolated vertices do not affect line graphs). Since *H* is 𝔇-free and connected, *F* has no triangles by Lemma 4.1. Since *F* has edges (which it does since *H* exists), *α*(*F̅*) ≤ 2. Moreover, as *F* is connected, *F̅* has no universal vertices, and so *i*(*F̅*) ≥ 2. Thus, *i*(*F̅*) = *α*(*F̅*) and *F̅* is well-covered. It follows that every edge of *F* corresponds to an *i*-set of *F̅*. Since *H* is the line graph of *F*, it is the *i*-graph of *F̅*.

Finally, if we examine Beineke’s forbidden subgraph characterization of line graphs (see [1] or [3, Theorem 6.26]), we note that eight of the nine minimal non-line graphs contain an induced 𝔇 and are therefore not *i*-graphs. The ninth minimal non-line graph is the claw, *K*_1,3_. Thus, 𝔇-free claw-free graphs are 𝔇-free line graphs, hence *i*-graphs.

**Corollary 4.3.**

*Let H be a connected claw-free graph. Then H is an i-graph if and only if H is* 𝔇-*free.*

While Theorem 4.2 and Corollary 4.3 reveal the *i*-graph realizability of many famous graph families (including another construction for cycles, which are connected, claw-free, and 𝔇-free), the realizability problem for graphs containing claws remains unresolved. Moreover, among clawed graphs are the theta graphs which we first alluded to in Section 2 as containing three of the small known non-*i*-graphs. In the next section we apply similar techniques with graph complements to construct all theta graphs that are *i*-graphs.

## 5. Theta graphs from graph complements

Consider a graph *G* with *i*(*G*) = 3. Each *i*-set of *G* is represented as a triangle (a *K*_3_) in *G̅*. If *X* and *Y* are two *i*-sets of *G* with $X\overset{\mathrm{uv}}{∼}Y$ , then *G̅*[*X*] and *G̅*[*Y*] are triangles in *G̅*, and have |*X* ∩ *Y*| = 2. Although it is technically the induced subgraphs *G̅*[*X*] and *G̅*[*Y* ] that are the triangles of *G̅*, for notational simplicity we refer to *X* and *Y* as triangles. In *G̅*, the triangle *X* can be transformed into the triangle *Y* by removing the vertex *u* and adding in the vertex *v* (where $u{≁}_{\bar{G}}v$ ). Thus, we say that two triangles are *adjacent* if they share exactly one edge. Moreover, since two *i*-sets of a graph *G* with *i*(*G*) = 3 are adjacent if and only if their associated triangles in *G̅* are adjacent, we use the same notation for *i*-set adjacency in *G* as triangle adjacency in *G̅*; that is, the notation *X* ∼ *Y* represents both *i*-sets *X* and *Y* of *G* being adjacent, and triangles *X* and *Y* of *G̅* being adjacent.

In the following sections we use triangle adjacency to construct *complement seed graphs* for the *i*-graphs that are theta graphs; that is, a graph *G̅* such that *ℐ* (*G*) is isomorphic to some desired theta graph. Before proceeding with these constructions, we note some observations which will help us with this process.

**Observation 5.1.**

*A graph G has i*(*G*) = 2 *if and only if G̅ is nonempty and has an edge that does not lie on a triangle.*

If *G̅* has an edge *uv* that does not lie on a triangle, then {*u, v*} is independent and dominating in *G*, and so *i*(*G*) ≤ 2. When building our seed graphs *G* with *i*(*G*) = 3, it is therefore necessary to ensure that every edge of the complement *G̅* belongs to a triangle.

**Observation 5.2.**

*Let G be a graph with i*(*G*) = 3. *If S with* |*S*| ≥ 4 *is a (possibly non-maximal) clique in G̅, then no* 3-*subset of S is an i-set of G.*

Suppose that *S* = {*u, v, w, x*} is such a clique of *G̅*. Then, for example, *x* is undominated by {*u, v, w*} in *G*, and so {*u, v, w*} is not an *i*-set of *G*. Conversely, suppose that {*u, v, w*} is a triangle in a graph *G̅* with *i*(*G*) = 3. By attaching a new vertex *x* to all of {*u, v, w*} in *G̅*, we remove {*u, v, w*} as an *i*-set of *G*, while keeping all other *i*-sets of *G*. This observation proves to be very useful in the constructions in the following section: we now have a technique to eliminate any unwanted triangles in *G̅* (and hence *i*-sets of *G*) that may arise. Note that this technique is an application of the Deletion Lemma (Lemma 3.10) in *G̅* instead of in *G*.

The use of triangle adjacency in a graph *G̅* to determine *i*-set adjacency in *G* provides a key technique to resolve the question of which theta graphs are *i-*graphs. In our main result, Theorem 5.3, we show that all theta graphs except the seven listed exceptions are *i*-graphs. Using this method of complement triangles, the proofs of the lemmas for the affirmative cases make up most of the remainder of Section 5. The proofs of the lemmas for the seven negative cases are given in Section 6.

**Theorem 5.3.**

*A theta graph is an i-graph if and only if it is not one of the seven exceptions listed below:*

$$\begin{array}{ccccc} \Theta 〈1,2,2〉, & & & & \\ \Theta 〈2,2,2〉, & \Theta 〈2,2,3〉, & \Theta 〈2,2,4〉, & \Theta 〈2,3,3〉, & \Theta 〈2,3,4〉,\\ \Theta 〈3,3,3〉. & & & & \end{array}$$

Table 1 summarizes the cases used to establish Theorem 5.3 and their associated results.

**Table 1.** *i*-graph realizability of theta graphs

Θ 〈*j, k, ℓ*〉	Realizability	Result

Θ 〈1, 2, 2〉	non-*i*-graph	𝔇. Proposition 3.7
Θ 〈1, 2, *ℓ*〉, *ℓ* ≥ 3	*i*-graph	Lemma 5.4
Θ 〈1, *k*, *ℓ*〉, 3 ≤ *k* ≤ *ℓ*	*i*-graph	Lemma 5.6
Θ 〈2, 2, 2〉	non-*i*-graph	*K*_2,3_. Proposition 3.7
Θ 〈2, 2, 3〉	non-*i*-graph	*κ*. Proposition 3.7
Θ 〈2, 2, 4〉	non-*i*-graph	Proposition 6.1
Θ 〈2, 2, *ℓ*〉, *ℓ* ≥ 5	*i*-graph	Lemma 5.10
Θ 〈2, 3, 3〉	non-*i*-graph	Proposition 6.2
Θ 〈2, 3, 4〉	non-*i*-graph	Proposition 6.3
Θ 〈2, 3, *ℓ*〉, *ℓ* ≥ 5	*i*-graph	Lemma 5.12
Θ 〈2, 4, 4〉	*i*-graph	Lemma 5.14
Θ 〈2, *k,* 5〉, 4 ≤ *k* ≤ 5	*i*-graph	Lemma 5.16
Θ 〈2, *k*, *ℓ*〉, *k* ≥ 4, *ℓ* ≥ 6, *ℓ* ≥ *k*	*i*-graph	Lemma 5.18
Θ 〈3, 3, 3〉	non-*i*-graph	Proposition 6.4
Θ 〈3, 3, 4〉	*i*-graph	Lemma 5.19
Θ 〈3, 3, 5〉	*i*-graph	Lemma 5.21
Θ 〈3, 3, *ℓ*〉, *ℓ* ≥ 6	*i*-graph	Lemma 5.23
Θ 〈3, 4, 4〉	*i*-graph	Lemma 5.25
Θ 〈3, 4, *ℓ*〉, *ℓ* ≥ 5	*i*-graph	Lemma 5.27
Θ 〈3, 5, 5〉	*i*-graph	Lemma 5.29
Θ 〈4, 4, 4〉	*i*-graph	Lemma 5.31
Θ 〈*j, k,* 5〉, 4 ≤ *j* ≤ *k* ≤ 5.	*i*-graph	Lemma 5.33
Θ 〈*j, k, ℓ*〉, 3 ≤ *j* ≤ *k* ≤ *ℓ*, and *ℓ* ≥ 6.	*i*-graph	Lemma 5.35

### 5.1. Θ 〈1, *k*, *ℓ*〉

We have already seen that the house graph *ℋ* = Θ 〈1, 2, 3〉 is an *i*-graph (Proposition 3.8). We can further exploit previous results to see that all graphs Θ 〈1, 2, *ℓ*〉 for *ℓ* ≥ 3 are *i*-graphs by taking a cycle *C_n_* with *n* ≥ 4, and replacing one of its maximal cliques (i.e. an edge) with a *K*_3_. By the Max Clique Replacement Lemma (Lemma 3.9), the resultant Θ 〈1, 2, *n −* 1〉 is also an *i-*graph. For reference, we explicitly state this result as a lemma.

**Lemma 5.4.**

*For ℓ* ≥ 3, *the theta graph* Θ 〈1, 2, *ℓ*〉 *is an i-graph.*

#### 5.1.1. Construction of *G̅* for Θ 〈1, *k*, *ℓ*〉, 3 ≤ *k* ≤ *ℓ*.

**Figure 5.**
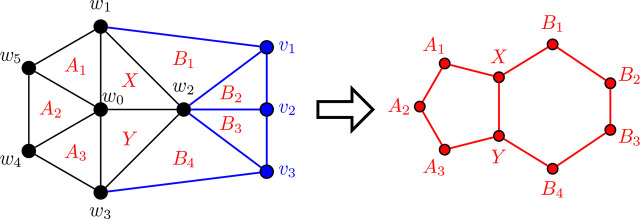
A graph *G̅* (left) such that *ℐ* (*G*) = Θ 〈1, 4, 5〉 (right)

In Figure 5 below, we provide a first example of the technique we employ repeatedly throughout this section to construct our theta graphs. To the left is a graph *G̅*, where each of its nine triangles corresponds to an *i*-set of its complement *G*. The resultant *i*-graph of *G*, *ℐ* (*G*) = Θ 〈1, 4, 5〉, is presented on the right. For consistency, we use *X* and *Y* to denote the triangles corresponding to the degree 3 vertices in the theta graphs in this example, as well as all constructions to follow.

We proceed now to the general construction of a graph *G̅* with *ℐ* (*G*) = Θ 〈1, *k*, *ℓ*〉 for 3 ≤ *k* ≤ *ℓ*. As it is our first construction using this triangle technique, we provide the construction and proof for Lemma 5.6 with an abundance of detail.

**Figure 6.**
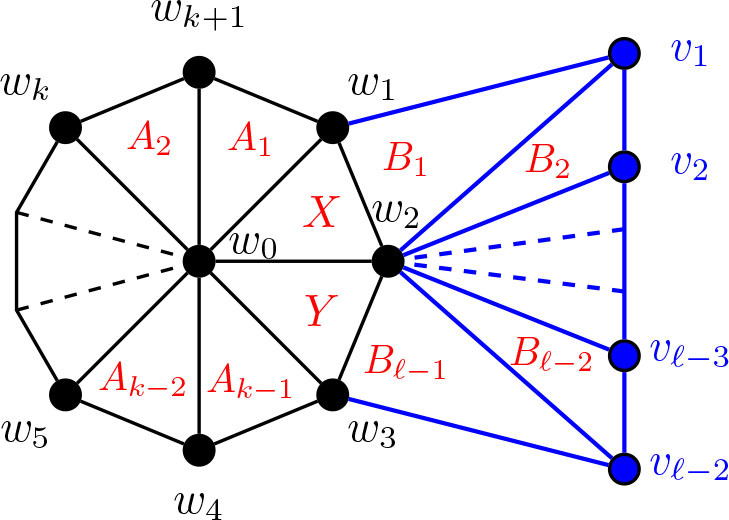
The graph *G̅* from Construction 5.5 such that *ℐ* (*G*) = Θ 〈1, *k*, *ℓ*〉 for 3 ≤ *k* ≤ *ℓ*

**Construction 5.5 (See Figure 6).**

Let *H̅* ≅ *W_k_*_+2_ = *C_k_*_+1_ ∨ *K*_1_, where *C_k_*_+1_ = (*w*_1_, . . . , *w_k_*_+1_, *w*_1_) and *w*_0_ is the central hub, i.e., the vertex with degree *k* + 1. Add a path *P_ℓ−_*_2_ : (*v*_1_, . . . , *v_ℓ−_*_2_), joining each *v_i_, i* = 1, ..., *ℓ −* 2, to *w*_2_. Join *v*_1_ to *w*_1_ and *v_ℓ−_*_2_ to *w*_3_. (If *ℓ* = 3, then *v*_1_ = *v_ℓ−_*_2_, hence *v*_1_ is adjacent to *w*_1_, *w*_2_ and *w*_3_.) This is the (planar) graph *G̅*.

**Lemma 5.6.**

*If G̅ is the graph constructed by Construction* 5.5, *then ℐ* (*G*) = *𝒜* (*G*) = Θ 〈1, *k*, *ℓ*〉, 3 ≤ *k* ≤ *ℓ.*

**Proof**

To begin, notice that since in *G̅*, the vertices *𝒲* = {*w*_0_, *w*_1_, . . . , *w_k_, w_k_*_+1_} form a wheel on at least five vertices, *H̅* ≆ *K*_4_. Likewise, the graph induced by {*w*_0_, *w*_1_, *w*_2_, *w*_3_, *v*_1_, *v*_2_, . . . , *v_ℓ−_*_2_} in *G̅* is also a wheel on *ℓ* + 2 vertices, where *w*_2_ is the central hub, and so it too contains no *K*_4_. Therefore, *G̅* is *K*_4_-free and the triangles of *G̅* are precisely the maximal cliques of *G̅*, and so *ω*(*G̅*) = *i*(*G*) = *α*(*G*) = 3. Since the *i*-sets of *G* are identical to its *α*-sets, *ℐ* (*G*) = *𝒜* (*G*), and so for ease of notation, we will refer only to *ℐ* (*G*) throughout the remainder of this proof.

We label the triangles as in Figure 6 by dividing them into two collections. The first are the triangles composed only of the vertices from *𝒲* and each containing *w*_0_: let *X* = {*w*_0_, *w*_1_, *w*_2_}, *Y* = {*w*_0_, *w*_2_, *w*_3_}, *A*_1_ = {*w*_0_, *w_k_*_+1_, *w*_1_}, *A*_2_ = {*w*_0_, *w_k_, w_k_*_+1_}, . . . , *A_k−_*_2_ = {*w*_0_, *w*_4_, *w*_5_}, *A_k−_*_1_ = {*w*_0_, *w*_3_, *w*_4_}. The remainder are the triangles with vertex sets not fully contained in *H̅*: *B*_1_ = {*w*_2_, *w*_1_, *v*_1_}, *B*_2_ = {*w*_2_, *v*_1_, *v*_2_}, *B*_3_ = {*w*_2_, *v*_2_, *v*_3_}, . . . , *B_ℓ−_*_2_ = {*w*_2_, *v_ℓ−_*_3_, *v_ℓ−_*_2_}, and *B_ℓ−_*_1_ = {*w*_2_, *v_ℓ−_*_2_, *w*_3_}. We refer to these collections as *𝒮* = {*X, Y* }, *𝒜* = {*A*_1_, *A*_2_, . . . , *A_k−_*_1_}, and *ℬ* = {*B*_1_, *B*_2_, . . . , *B_ℓ−_*_1_}. It is clear from Figure 6 that these are the only triangles of *G̅*. Therefore *V* (*ℐ* (*G*)) = {*X, Y, A*_1_, *A*_2_, . . . , *A_k−_*_1_, *B*_1_, *B*_2_, . . . , *B_ℓ−_*_1_}.

We now show that the required adjacencies hold. From the construction of *G̅*, the following are immediate for *ℐ* (*G*):

(i)
$X\overset{w_{1}w_{3}}{∼}Y$ ,
(ii)
$X\overset{w_{0}v_{1}}{∼}B_{1}\overset{w_{1}v_{2}}{∼}B_{2}\overset{v_{1}v_{3}}{∼}B_{3}\ldots B_{\ell -2}\overset{v_{\ell -3}w_{3}}{∼}B_{\ell -1}\overset{v_{\ell -2}w_{0}}{∼}Y$ ,
(iii)
$X\overset{w_{2}w_{k+1}}{∼}A_{1}\overset{w_{1}w_{k}}{∼}A_{2}\ldots A_{k-2}\overset{w_{5}w_{3}}{∼}A_{k-1}\overset{w_{4}w_{2}}{∼}Y$ .

Hence, we need only show that there are no additional unwanted edges generated in the construction of *ℐ* (*G*).

Since *G̅* is a planar graph and all of its triangles are facial (that is, the edges of the *K*_3_ form a face in the plane embedding in Figure 6), each triangle is adjacent to at most three others. From (i)–(iiit287) above, triangles *X* and *Y* are both adjacent to the maximum three (and hence deg_*ℐ* (*G*)_(*X*) = deg_*ℐ* (*G*)_(*Y*) = 3).

Recall that to be adjacent, two triangles share exactly two vertices. Notice that the triangles of *𝒜* are composed entirely of vertices from *𝒲 −* {*w*_2_}, and that for 2 ≤ *i* ≤ *ℓ −* 2, *B_i_* ∩ *𝒲* = {*w*_2_}; furthermore, *B*_1_ ∩ *𝒲* = {*w*_1_, *w*_2_} and *B_ℓ−_*_2_ ∩ *𝒲* = {*w*_2_, *w*_3_}. Therefore no triangle of *ℬ* is adjacent to any triangle of *𝒜*. It is similarly easy to see that there are no additional unwanted adjacencies between two triangles of *𝒜* or two triangles of *ℬ*.

We conclude that the graph *G̅* generated by Construction 5.5 yields *ℐ* (*G*) = *𝒜* (*G*) = Θ 〈1, *k*, *ℓ*〉.

**Figure 7.**
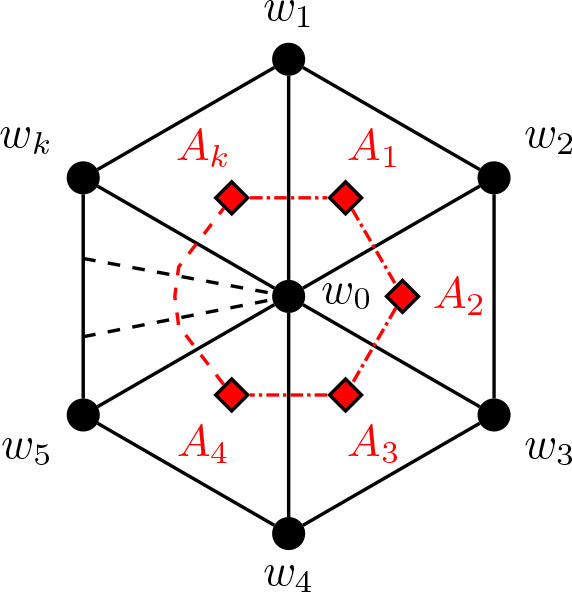
The wheel *H_k_* = *W_k_*_+1_, with the *i*-graph of its complement, $ℐ\left(\bar{H_{k}}\right)≅𝒜\left(\bar{H_{k}}\right)≅C_{k}$ , embedded in red

Before we proceed with the remainder of the theta graph constructions, let us return to Figure 6 to notice the prominence of the wheel subgraph in the complement seed graph *G̅*. In the constructions throughout this paper, this wheel subgraph will appear repeatedly; indeed, all of the complement seed graphs for the *i*-graphs of theta graphs have a similar basic form: begin with a wheel, add a path of some length, and then add some collection of edges between them. As stated without proof in Lemma 5.7, Figure 7 below demonstrates that a wheel in a triangle-based complement seed graph *G̅* corresponds to a cycle in the *i*-graph of *G*. Using this result, in our later constructions with a wheel subgraph, we already have two of the three paths of a theta graph formed. Hence, we need only confirm that whatever unique additions are present in a given construction form the third path in the *i*-graph.

**Lemma 5.7.**

*For k* ≥ 4, *let H_k_ be the wheel W_k_*_+1_ = *C_k_* ∨ *K*_1_. *Then* $ℐ\left(\bar{H_{k}}\right)≅𝒜\left(\bar{H_{k}}\right)≅C_{k}$ .

**Figure 8.**
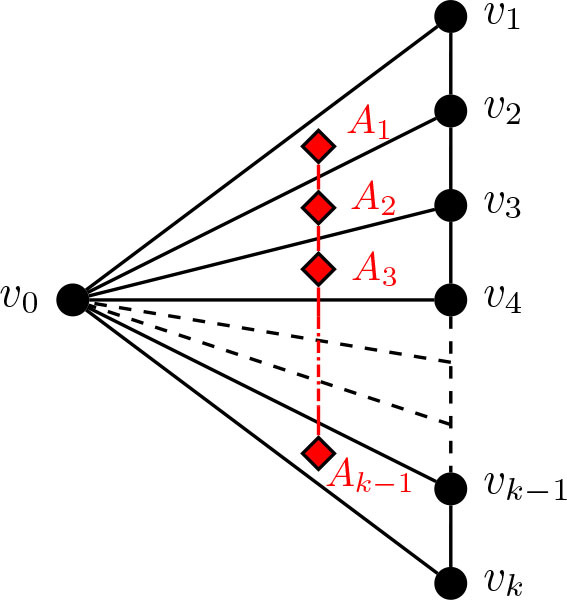
The fan *H_k_* = *K*_1_ ∨ *P_k_*, with the *i*-graph of its complement, $ℐ\left(\bar{H_{k}}\right)≅𝒜\left(\bar{H_{k}}\right)≅P_{k-1}$ , embedded in red

We note the following analogous – although less frequently applied – result for fans of the form *K*_1_ + *P_k_*, as illustrated in Figure 8.

**Lemma 5.8.**

*For k* ≥ 2, *let H_k_ be the k-fan K*_1_ ∨ *P_k_. Then* $ℐ\left(\bar{H_{k}}\right)≅𝒜\left(\bar{H_{k}}\right)≅P_{k-1}$ .

### 5.2. Θ 〈2, *k*, *ℓ*〉 for 2 ≤ *k* ≤ *ℓ*

#### 5.2.1. Construction of *G̅* for Θ 〈2, 2, *ℓ*〉, ℓ ≥ 5

As stated in Proposition 3.7, *K*_2,3_ ≅ Θ 〈2, 2, 2〉 and *κ* ≅ Θ 〈2, 2, 3〉 are not *i*-graphs. Extending these results, we find that the length of the third path in Θ 〈2, 2, *ℓ*〉 has a transition point between *ℓ* = 4 and *ℓ* = 5; while *ℓ* = 4 is still too short to form an *i*-graph (see Lemma 6.1), for *ℓ* ≥ 5, Θ 〈2, 2, *ℓ*〉 is *i*-graph realizable.

**Construction 5.9.**

**Figure 9.**
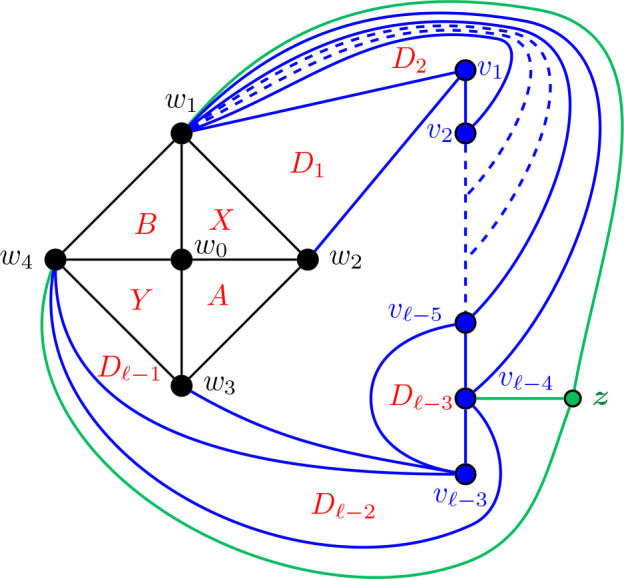
The graph *G̅* from Construction 5.9 such that *ℐ* (*G*) = Θ 〈2, 2, *ℓ*〉 for *ℓ* ≥ 6

**Figure 10.**
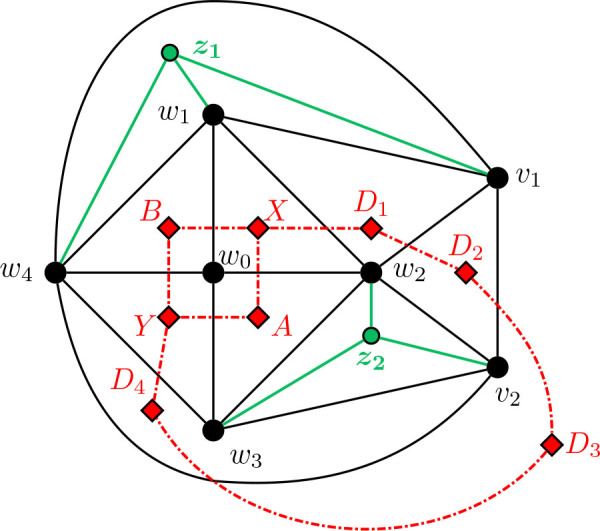
The graph *G̅* from Construction 5.9 such that *ℐ* (*G*) = Θ 〈2, 2, 5〉, with Θ 〈2, 2, 5〉 overlaid in red

See Figures 9 and 10. Begin with the graph *H̅* ≅ *W*_5_ = *C*_4_ ∨ *K*_1_, labelling the degree 3 vertices as *w*_1_, *w*_2_, *w*_3_, *w*_4_ and the central degree 4 vertex as *w*_0_.

(a) If *ℓ* ≥ 6 (as in Figure 9), attach to *H̅* a path *P_ℓ−_*_3_ : (*v*_1_, *v*_2_, . . . , *v_ℓ−_*_3_) by joining *w*_1_ to *v*_1_, *v*_2_, . . . , *v_ℓ−_*_4_. Join *v_ℓ−_*_5_ to *v_ℓ−_*_3_. Next, join *w*_2_ to *v*_1_, and *w*_3_ to *v_ℓ−_*_3_. Then, join *w*_4_ to *v_ℓ−_*_4_ and *v_ℓ−_*_3_. Add a new vertex *z*, joined to *w*_1_, *w*_4_, and *v_ℓ−_*_4_.
(b) If *ℓ* = 5 (as in Figure 10), attach to *H̅* a path of *P*_2_ : (*v*_1_, *v*_2_), by joining *w*_1_ to *v*_1_, and *w*_2_ to *v*_1_ and *v*_2_. Then join *w*_3_ to *v*_2_, and *w*_4_ to both *v*_1_ and *v*_2_. Add two new vertices, *z*_1_ and *z*_2_, joining *z*_1_ to *v*_1_, *w*_1_, and *w*_4_, and *z*_2_ to *v*_2_, *w*_2_ and *w*_3_.

We label the resultant (planar) graph *G̅*.

As with our other constructions, the triangles of *G̅* are its smallest maximal cliques, and so *i*(*G*) = 3. However, we now employ a technique of adding vertices to create *K*_4_’s in *G̅* and eliminate any “unwanted” triangles that might arise in our construction. In (b), the addition of *z*_1_ and *z*_2_ eliminate triangles {*w*_1_, *w*_4_, *v*_1_} and {*w*_2_, *w*_3_, *v*_2_}, respectively. Similarly, in (a), *z* prevents {*w*_1_, *w*_4_, *v_ℓ−_*_3_} from being a maximal clique of *G̅* and hence an *i*-set of *G*. The unfortunate trade-off in this triangle-elimination technique is that the remaining triangles are no longer *α*-sets; the constructions work only for *i*-graphs, not *α*-graphs.

**Lemma 5.10.**

*If G̅ is the graph constructed by Construction* 5.9, *then ℐ* (*G*) = Θ 〈2, 2, *ℓ*〉, *for ℓ* ≥ 5.

**Proof**

We only prove the lemma for case where *ℓ* ≥ 6; the single case where *ℓ* = 5 is adequately illustrated in Figure 10 and details can be found in [9].

As in the previous constructions, since each edge of *G̅* belongs to a triangle and some triangles are not contained in *K*_4_’s, these triangles of *G̅* form the smallest maximal cliques of *G̅*. We label these triangles as in Figure 9; in particular,

$$\begin{array}{lll} X & = & \left\{w_{0},\;w_{1},\;w_{2}\right\},\\ Y & = & \left\{w_{0},\;w_{3},\;w_{4}\right\},\\ A & = & \left\{w_{0},\;w_{2},\;w_{3}\right\},\\ B & = & \left\{w_{0},\;w_{1},\;w_{4}\right\},\\ D_{1} & = & \left\{w_{1},\;w_{2},\;v_{1}\right\},\\ D_{i} & = & \left\{w_{1},\;v_{i-1},\;v_{i}\right\}\;\;\;\;\text{for}\;2\leq i\leq \ell -4,\\ D_{\ell -3} & = & \left\{v_{\ell -5},\;v_{\ell -4},\;v_{\ell -3}\right\},\\ D_{\ell -2} & = & \left\{w_{4},\;v_{\ell -4},\;v_{\ell -3}\right\},\\ D_{\ell -1} & = & \left\{w_{3},\;w_{4},\;v_{\ell -3}\right\}. \end{array}$$

It is straightforward to verify that these *ℓ*+3 sets are precisely the maximal cliques of *G̅* of order 3 and, hence, the *i*-sets of *G*. Therefore they form the vertex set of *ℐ* (*G*). Moving to the edges of *ℐ* (*G*), the following adjacencies are clear from Figure 9:

(i)
$X\overset{w_{1}w_{3}}{∼}A\overset{w_{2}w_{4}}{∼}Y$ ,
(ii)
$X\overset{w_{2}w_{4}}{∼}B\overset{w_{1}w_{3}}{∼}Y$ ,
(iii)
$X\overset{w_{0}v_{1}}{∼}D_{1}\overset{w_{2}v_{2}}{∼}D_{2}\overset{v_{1}v_{3}}{∼}D_{3}\ldots D_{\ell -3}\overset{w_{1}v_{\ell -2}}{∼}D_{\ell -2}\overset{v_{\ell -4}w_{4}}{∼}D_{\ell -1}\overset{v_{\ell -2}w_{0}}{∼}Y$ .

As for its vertex set, it is straightforward to verify that the edge set of *ℐ* (*G*) consists of precisely the edges listed in (i)–(iii). We conclude that *ℐ* (*G*) = Θ 〈2, 2, *ℓ*〉.

#### 5.2.2. Construction of *G̅* for Θ 〈2, 3, *ℓ*〉 for *ℓ* ≥ 5

For many of the results going forward, we apply small modifications to previous constructions. In the first of these, we begin with the graphs from Construction 5.9, which were used to find *i*-graphs for Θ 〈2, 2, 5〉 and Θ 〈2, 2, *ℓ*〉 for *ℓ* ≥ 6, and expand the central wheel used in there to build *i*-graphs for Θ 〈2, 3, 5〉 and Θ 〈2, 3, *ℓ*〉 for *ℓ* ≥ 6.

**Construction 5.11.**

**Figure 11.**
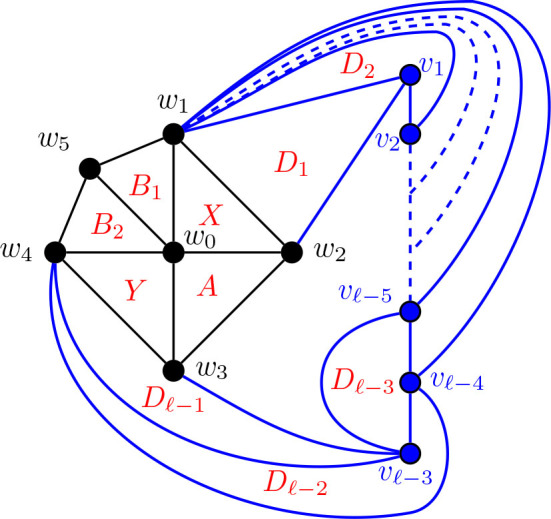
The graph $\bar{G_{2,3,\ell }}$ from Construction 5.11 (a) such that *ℐ* (*G*_2,3, *ℓ*_) = *𝒜* (*G*_2,3, *ℓ*_) = Θ 〈2, 3, *ℓ*〉 for *ℓ* ≥ 6

**Figure 12.**
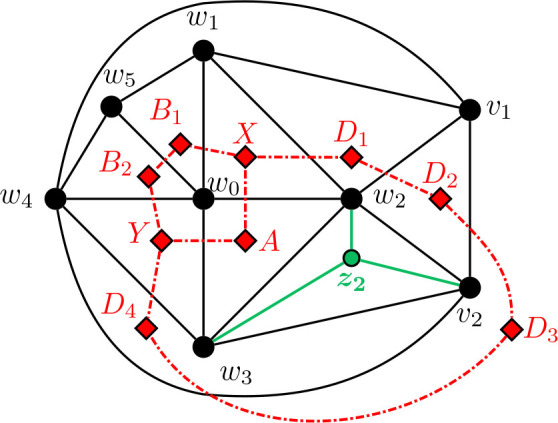
The graph $\bar{G_{2,3,5}}$ from Construction 5.11 (b) such that *ℐ* (*G*_2,3,5_) = Θ 〈2, 3, 5〉, with *ℐ* (*G*_2,3,5_) over-laid in red

Refer to Figures 11 and 12.

(a) If *ℓ* ≥ 6, begin with a copy of the graph *G̅* from Construction 5.9. Subdivide the edge *w*_1_*w*_4_, adding the new vertex *w*_5_. Join *w*_5_ to *w*_0_, so that *w*_0_, *w*_1_, . . . , *w*_5_ forms a wheel. Delete the vertex *z*.
(b) If *ℓ* = 5, begin with a copy of the graph *G̅* from Construction 5.9. Subdivide the edge *w*_1_*w*_4_, adding the new vertex *w*_5_. Join *w*_5_ to *w*_0_, so that *w*_0_, *w*_1_, . . . , *w*_5_ forms a wheel. Delete the vertex *z*_1_.

We rename the resultant (planar) graph $\bar{G_{2,3,\ell }}$ for *ℓ* ≥ 5.

In Construction 5.11 (a), notice that the vertex *z* is deleted from *G̅*. In the original Construction 5.9 for a graph *G̅* with *ℐ* (*G*) = Θ 〈2, 2, *ℓ*〉 for *ℓ* ≥ 6, *z* served to eliminate the unwanted triangle formed by {*w*_1_, *w*_4_, *v_ℓ−_*_4_}. Now with the expanded wheel including *w*_5_, {*w*_1_, *w*_4_, *v_ℓ−_*_4_} is not a triangle in $\bar{G_{2,3,\ell }}\left(\ell \geq 6\right)$ , and *z* is not needed. Indeed, as $\bar{G_{2,3,\ell }}$ now has *α*(*G*_2,3, *ℓ*_) = 3, its triangles are also *α*-sets in *G*, and so we can immediately extend the construction from *i*-graphs to *α*-graphs.

The extension, however, does not apply to Construction 5.11 (b) for the graph $\bar{G_{2,3,5}}$ . Here, we no longer require *z*_1_ (which served to eliminate the unwanted triangle formed by {*w*_1_, *w*_4_, *v*_1_}), but *z*_2_ remains and forms the clique {*w*_2_, *w*_3_, *z*_2_, *v*_2_}; thus, *α*(*G*_2,3,5_) = 4.

The proof for the following Lemma 5.12 is otherwise very similar to the proof of Lemma 5.10, and so is omitted.

**Lemma 5.12.**

*If* $\bar{G_{2,3,5}}$ *is the graph constructed in Construction* 5.11 (b), *then ℐ* (*G*_2,3,5_) = Θ 〈2, 3, 5〉. *For ℓ* ≥ 6, *if* $\bar{G_{2,3,\ell }}$ *is the graph constructed in Construction* 5.11 (a), *then ℐ* (*G*_2,3, *ℓ*_) = *𝒜* (*G*_2,3, *ℓ*_) = Θ 〈2, 3, *ℓ*〉 *for ℓ* ≥ 6.

#### 5.2.3. Construction of *G̅* for Θ 〈2, 4, 4〉

In the following construction for a graph *G̅* with *ℐ* (*G*) = Θ 〈2, 4, 4〉, we again apply the technique of adding vertices to eliminate unwanted triangles.

**Figure 13.**
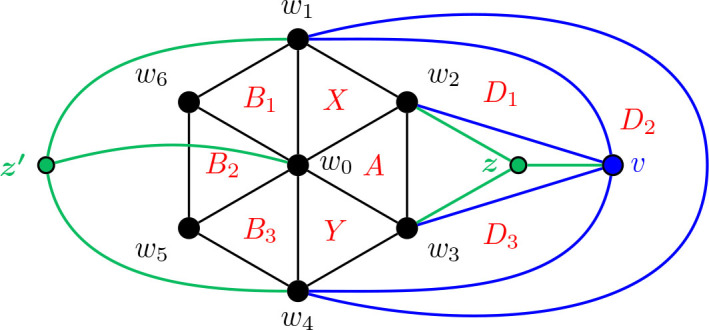
A graph *G̅* such that *ℐ* (*G*) = Θ 〈2, 4, 4〉

**Construction 5.13.**

Refer to Figure 13. Begin with a copy of the graph *H̅* ≅ *W*_7_ = *C*_6_ ∨ *K*_1_, labelling the degree 3 vertices as *w*_1_, *w*_2_, . . . , *w*_6_ and the central degree 6 vertex as *w*_0_. Join *w*_1_ to *w*_4_. Add a new vertex *v* to *H̅*, joining *v* to *w*_1_, . . . , *w*_4_. Then, add the new vertex *z*, joined to *v*, *w*_2_ and *w*_3_, and the new vertex *z′*, joined to *w*_0_, *w*_1_, and *w*_4_. We label the resultant (non-planar) graph *G̅*.

**Lemma 5.14.**

*If G̅ is the graph constructed by Construction* 5.13, *then ℐ* (*G*) = Θ 〈2, 4, 4〉.

**Proof**

As shown in Figure 13, the triangles of *G̅* forming maximal cliques of *G̅*, and therefore the *i*-sets of *G*, are labelled them as follows:

$$\begin{array}{lll} X=\left\{w_{0},w_{1},w_{2}\right\}, & B_{1}=\left\{w_{0},w_{1},w_{6}\right\}, & D_{1}=\left\{w_{1},\;w_{2},\;v\right\},\\ Y=\left\{w_{0},w_{3},w_{4}\right\}, & B_{2}=\left\{w_{0},w_{5},w_{6}\right\} & D_{2}=\left\{w_{1},\;w_{4},\;v\right\},\\ A=\left\{w_{0},w_{2},w_{3}\right\}, & B_{3}=\left\{w_{0},w_{4},w_{5}\right\}, & D_{3}=\left\{w_{3},\;w_{4},\;v\right\}. \end{array}$$

Similarly to the construction for Θ 〈2, 2, 5〉 in the proof of Lemma 5.10, the vertices *z* and *z′* are added to ensure that {*v, w*_2_, *w*_3_} and {*w*_0_, *w*_1_, *w*_4_}, respectively, are not maximal cliques in *G̅*, and hence are not *i*-sets of *G*.

It can be seen that there are no other triangles in *G̅* beyond the nine listed above; we omit the details, which can be found in [9, Lemma 5.19].

From Figure 13, the following triangle adjacencies are immediate:

(i)
$X\overset{w_{1}w_{3}}{∼}A\overset{w_{2}w_{4}}{∼}Y$ ,
(ii)
$X\overset{w_{2}w_{6}}{∼}B_{1}\overset{w_{1}w_{5}}{∼}B_{2}\overset{w_{6}w_{4}}{∼}B_{3}\overset{w_{5}w_{3}}{∼}Y$ ,
(iii)
$X\overset{w_{0}v}{∼}D_{1}\overset{w_{2}w_{4}}{∼}D_{2}\overset{w_{1}w_{3}}{∼}D_{3}\overset{vw_{0}}{∼}Y$ .

It is again straightforward to verify that there are no additional edges in *ℐ* (*G*) than those listed above. We conclude that *ℐ* (*G*) = Θ 〈2, 4, 4〉 as required.

Notice that Construction 5.13 is our first theta graph construction that is not planar as it has a *K*_3,3_ minor. Indeed, with the exception of the constructions that are based upon Construction 5.13, all of our *i*-graph constructions use planar complement seed graphs.

**Problem 1.**

(i) Find a planar graph-complement construction for Θ 〈2, 4, 4〉.
(ii) Do all *i*-graphs with largest induced stars of *K*_1,3_, always have a planar graph-complement construction?
  A large target graph requires a large seed graph in order to generate a sufficient number of unique *i*-sets. Can a target graph become too dense to allow for a planar graph-complement construction?

Moving forward, we will no longer explicitly check that there are no additional unaccounted for triangles in our constructions. Should the construction indeed result in triangles of *G̅* that produce extraneous vertices in *ℐ* (*G*), we can easily remove them using the Deletion Lemma (Lemma 3.10).

#### 5.2.4. Constructions of *G̅* for Θ 〈2, 4, 5〉 and Θ 〈2, 5, 5〉

**Construction 5.15.**

Refer to Figure 14.

(a) Begin with a copy of the graph $\bar{G_{2,3,5}}$ from Construction 5.11 (b) for Θ 〈2, 3, 5〉. Subdivide the edge *w*_1_*w*_5_, adding the new vertex *w*_6_. Join *w*_6_ to *w*_0_, so that *w*_0_, *w*_1_, . . . , *w*_6_ forms a wheel. Call this graph $\bar{G_{2,4,5}}$ .
(b) Begin with a copy of the graph $\bar{G_{2,4,5}}$ from Construction 5.15 (a). Subdivide the edge *w*_1_*w*_6_, adding the new vertex *w*_7_. Join *w*_7_ to *w*_0_, so that *w*_0_, *w*_1_, . . . , *w*_7_ forms a wheel. Call this graph $\bar{G_{2,5,5}}$ .

**Figure 14.**
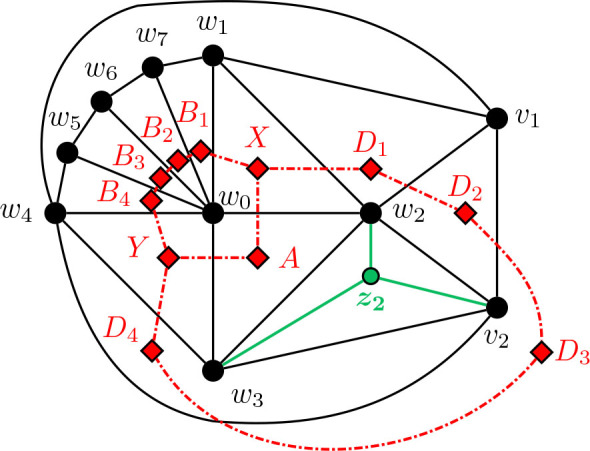
The graph $\bar{G_{2,5,5}}$ from Construction 5.15 such that *ℐ* (*G*) = Θ 〈2, 5, 5〉, with *ℐ* (*G*) overlaid in red.

**Lemma 5.16.**

*If* $\bar{G_{2,k,5}}$ *is the graph constructed by Construction* 5.15, *then ℐ* (*G*_2,*k*,5_) = Θ 〈2, *k,* 5〉, *for* 4 ≤ *k* ≤ 5.

Lemma 5.16 follows readily from Figure 14 and we omit the proof.

#### 5.2.5. Construction of *G̅* for Θ 〈2, *k*, *ℓ*〉 for *ℓ* ≥ *k* ≥ 4 and *ℓ* ≥ 6

**Construction 5.17.**

**Figure 15.**
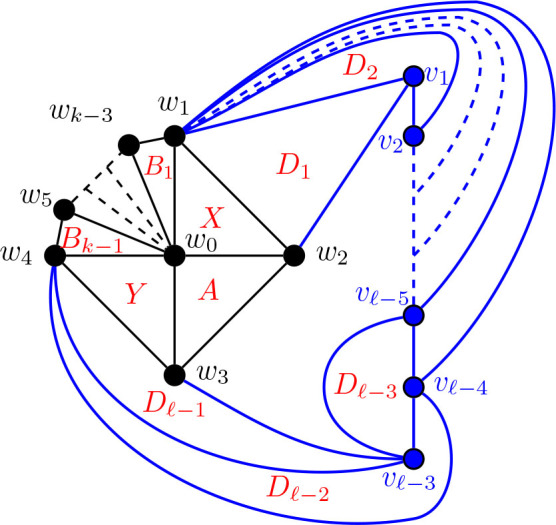
The graph $\bar{G_{2,k,\ell }}$ from Construction 5.17 such that $ℐ\left(\bar{G_{2,k,\ell }}\right)=\Theta 〈2,k,\ell 〉$ for *ℓ* ≥ *k* ≥ 4 and *ℓ* ≥ 6

See Figure 15. Begin with a copy of the graph $\bar{G_{2,3,\ell }}$ from Construction 5.11 (a) for *ℓ* ≥ 5. Subdivide the edge *w*_1_*w*_5_ *k −* 3 times (for *k* ≤ *ℓ*), adding the new vertices *w*_6_, *w*_7_, . . . , *w_k_*_+2_. Join *w*_6_, *w*_7_, . . . , *w_k_*_+2_ to *w*_0_, so that *w*_0_, *w*_1_, . . . , *w_k_*_+2_ forms a wheel. Call this graph $\bar{G_{2,k,\ell }}$ .

**Lemma 5.18.**

*If* $\bar{G_{2,k,\ell }}$ *is the graph constructed by Construction* 5.17, *then ℐ* (*G*_2,*k*,*ℓ*_) = *𝒜* (*G*_2,*k*,*ℓ*_) = Θ 〈2, *k*, *ℓ*〉, *for ℓ* ≥ *k* ≥ 4 *and ℓ* ≥ 6.

Lemma 5.18 follows from Figure 15.

### 5.3. Θ 〈3, *k*, *ℓ*〉

In the rest of Section 5 we only give the constructions and their associated figures, and state the lemmas without proof. Details can be found in [9, Chapter 5].

#### 5.3.1. The graph *G̅* for Θ 〈3, 3, 4〉

**Figure 16.**
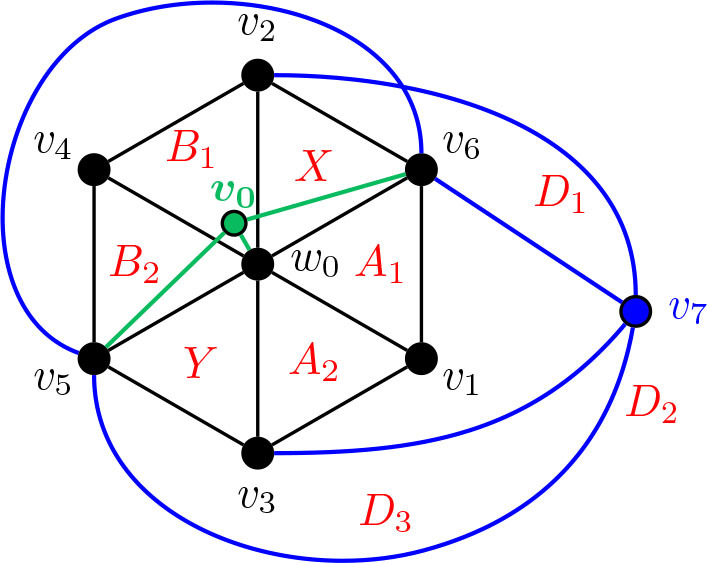
A graph *G̅* such that *ℐ* (*G*) = Θ 〈3, 3, 4〉

The triangles *X, A*_1_, *A*_2_, *Y, B*_2_, *B*_1_, *D*_1_, *D*_2_, *D*_3_ in Figure 16, and the obvious paths formed by them, illustrate the following lemma.

**Lemma 5.19.**

Θ 〈3, 3, 4〉 *is an i-graph.*

#### 5.3.2. Construction of G̅ for Θ 〈3, 3, 5〉

**Construction 5.20.**

**Figure 17.**
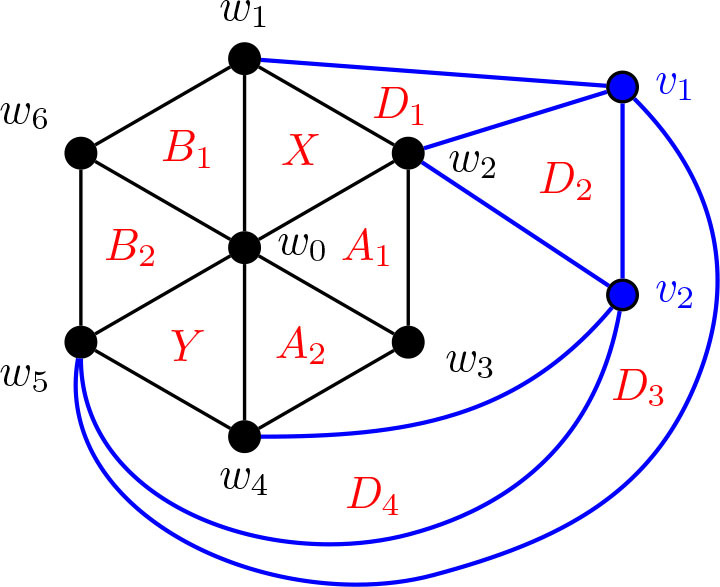
A graph $\bar{G_{3,3,5}}$ from Construction 5.20 such that *ℐ* (*G*_3,3,5_) = Θ 〈3, 3, 5〉.

Refer to Figure 17. Begin with a copy of the graph *H̅* ≅ *W*_7_ = *C*_6_ ∨ *K*_1_, labelling the degree 3 vertices as *w*_1_, *w*_2_, . . . , *w*_6_ and the central degree 6 vertex as *w*_0_. Add new vertices *v*_1_ and *v*_2_ to *H̅*, joined to each other. Then, join *v*_1_ to each of {*w*_1_, *w*_2_, *w*_5_}, and *v*_2_ to each of {*w*_2_, *w*_4_, *w*_5_}. Call this graph $\bar{G_{3,3,5}}$ .

**Lemma 5.21.**

*If* $\bar{G_{3,3,5}}$ *is the graph constructed by Construction* 5.20, *then ℐ* (*G*_3,3,5_) = *𝒜* (*G*_3,3,5_) = Θ 〈3, 3, 5〉.

#### 5.3.3. Construction of *G̅* for Θ 〈3, 3, *ℓ*〉 for *ℓ* ≥ 6

**Construction 5.22.**

Refer to Figure 19. Begin with a copy of the graph *H̅* ≅ *W*_7_ = *C*_6_ ∨ *K*_1_, labelling the degree 3 vertices as *w*_1_, *w*_2_, . . . , *w*_6_ and the central degree 4 vertex as *w*_0_. For *ℓ* ≥ 6, add to *H̅* a new path of *ℓ −* 3 vertices labelled as *v*_1_, *v*_2_, . . . , *v_ℓ−_*_3_. Then, join *w*_1_ to each of {*v*_1_, *v*_2_, . . . , *v_ℓ−_*_4_}, *w*_4_ to *v_ℓ−_*_3_, and *w*_5_ to *v_ℓ−_*_4_ and *v_ℓ−_*_3_. Finally, join *v_ℓ−_*_5_ to *v_ℓ−_*_3_, so that {*v_ℓ−_*_5_, *v_ℓ−_*_4_, *v_ℓ−_*_3_} form a *K*_3_. Call this graph $\bar{G_{3,3,\ell }}$ for *ℓ* ≥ 6.

Although the general construction still applies for the case when *ℓ* = 6, we include a separate figure for the construction of Θ 〈3, 3, 6〉 for reference below, because of the additional complication that now *v*_1_ = *v_ℓ−_*_5_, and so the single vertex now has dual roles in the construction.

**Lemma 5.23.**

*If* $\bar{G_{3,3,\ell }}$ *is the graph constructed by Construction* 5.22, *then ℐ* (*G*_3,3, *ℓ*_) = *𝒜* (*G*_3,3, *ℓ*_) = Θ 〈3, 3, *ℓ*〉 *for ℓ* ≥ 6.

**Figure 18.**
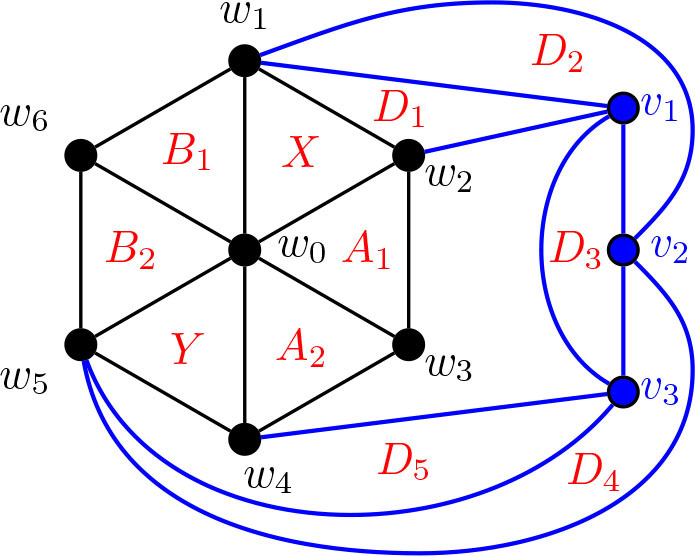
The graph $\bar{G_{3,3,6}}$ from Construction 5.22 such that *ℐ* (*G*_3,3,6_) = *𝒜* (*G*_3,3,6_) = Θ 〈3, 3, 6〉

**Figure 19.**
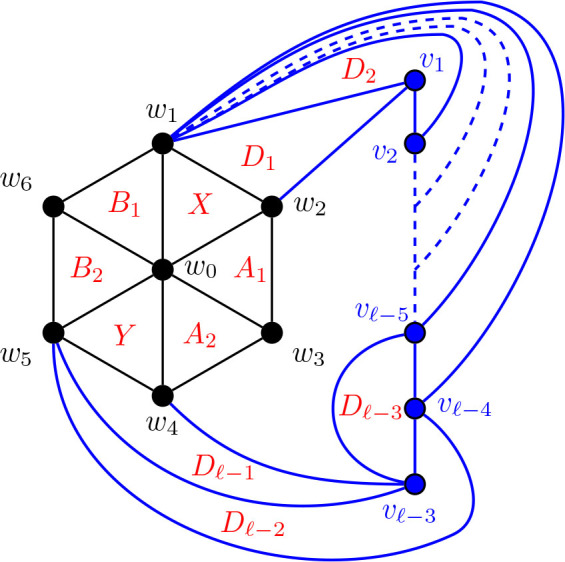
The graph $\bar{G_{3,3,\ell }}$ from Construction 5.22 such that *ℐ* (*G*) = *𝒜* (*G*) = Θ 〈3, 3, *ℓ*〉 for *ℓ* ≥ 6

#### 5.3.4. Construction of *G̅* for Θ 〈3, 4, 4〉

**Construction 5.24.**

**Figure 20.**
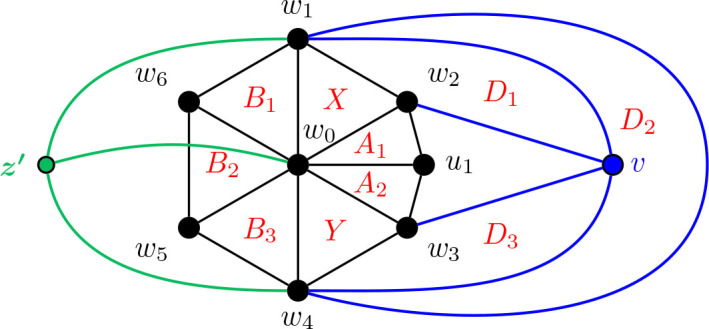
The graph $\bar{G_{3,4,4}}$ from Construction 5.24 such that *ℐ* (*G*_3,4,4_) = Θ 〈3, 4, 4〉

See Figure 20. Begin with a copy of the graph *G̅* from Construction 5.13 for Θ 〈2, 4, 4〉, which we rename here as $\bar{G_{2,4,4}}$ . Subdivide the edge *w*_2_*w*_3_, adding a new vertex *u*_1_, and joining *u*_1_ to *w*_0_. Delete the vertex *z*. Call this graph $\bar{G_{3,4,4}}$ .

**Lemma 5.25.**

*If* $\bar{G_{3,4,4}}$ *is the graph constructed by Construction* 5.24, *then ℐ* (*G*_3,4,4_) = Θ 〈3, 4, 4〉.

#### 5.3.5. Construction of *G̅* for Θ 〈3, 4, *ℓ*〉, *ℓ* ≥ 5

**Construction 5.26.**

Begin with a copy of the graph $\bar{G_{3,4,4}}$ from Construction 5.24 for Θ 〈3, 4, 4〉. Subdivide the edge *w*_1_*w*_6_ *ℓ −* 4 times (for *ℓ* ≥ 5), adding the new vertices *w*_7_, *w*_8_, . . . , *w_ℓ_*_+2_. Join *w*_7_, *w*_8_, . . . , *w_ℓ_*_+2_ to *w*_0_, so that *w*_0_, *w*_1_, . . . , *w_ℓ_*_+2_ forms a wheel. Call this graph $\bar{G_{3,4,\ell }}$ .

**Lemma 5.27.**

*If* $\bar{G_{3,4,\ell }}$ *is the graph constructed by Construction* 5.26, *then ℐ* (*G*_3,4, *ℓ*_) = Θ 〈3, 4, *ℓ*〉 *for ℓ* ≥ 5.

#### 5.3.6. Construction of *G̅* for Θ 〈3, 5, 5〉

**Construction 5.28.**

**Figure 21.**
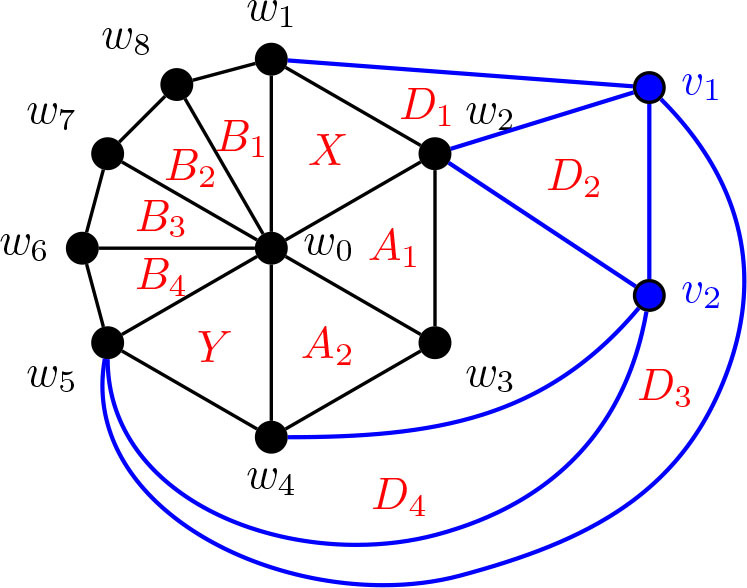
A graph $\bar{G_{3,5,5}}$ from Construction 5.28 such that *ℐ* (*G*_3,5,5_) = Θ 〈3, 5, 5〉.

Refer to Figure 21. Begin with a copy of the graph $\bar{G_{3,3,5}}$ from Construction 5.20. Subdivide the edge *w*_1_*w*_6_ twice, adding the new vertices *w*_7_ and *w*_8_. Join *w*_7_ and *w*_8_ to *w*_0_, so that *w*_0_, *w*_1_, . . . , *w*_8_ forms a wheel. Call this graph $\bar{G_{3,5,5}}$ .

**Lemma 5.29.**

*If* $\bar{G_{3,5,5}}$ *is the graph constructed by Construction* 5.28, *then ℐ* (*G*_3,5,5_) = *𝒜* (*G*_3,5,5_) = Θ 〈3, 5, 5〉.

Notice that subdividing only once in Construction 5.28 (adding only *w*_7_ and not *w*_8_) gives an alternative (planar) construction for Θ 〈3, 4, 5〉.

### 5.4. Θ 〈*j*, *k*, *ℓ*〉 for 4 ≤ *j* ≤ *k* ≤ *ℓ* and 3 ≤ *j* ≤ *k* ≤ *ℓ*, *ℓ* ≥ 6

#### 5.4.1. Construction of *G̅* for Θ 〈*j*, *k*, *ℓ*〉 for 4 ≤ *j* ≤ *k* ≤ *ℓ* ≥ 5

**Construction 5.30.**

**Figure 22.**
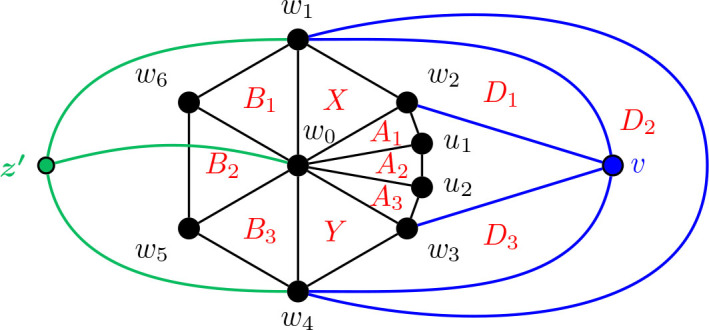
The graph $\bar{G_{4,4,4}}$ from Construction 5.30 such that *ℐ* (*G*_4,4,4_) = Θ 〈4, 4, 4〉

Refer to Figure 22. Begin with a copy of the graph $\bar{G_{3,4,4}}$ from Construction 5.24. Subdivide the edge *u*_1_*w*_3_, adding the new vertex *u*_2_. Join *u*_2_ to *w*_0_, so that

$$w_{0},w_{1},w_{2},u_{1},u_{2},w_{3},\ldots ,w_{6}$$

forms a wheel. Call this graph $\bar{G_{4,4,4}}$ .

**Lemma 5.31.**

*If* $\bar{G_{4,4,4}}$ *is the graph constructed by Construction* 5.30, *then ℐ* (*G*_4,4,4_) = Θ 〈4, 4, 4〉.

**Construction 5.32.**

Begin with a copy of the graph $\bar{G_{3,3,5}}$ from Construction 5.20. For *k* = 4 subdivide the edge *w*_1_*w*_6_ once, adding the vertex *w*_7_; for *k* = 5, subdivide a second time, adding the vertex *w*_8_. For *j* = 4, subdivide the edge *w*_2_*w*_3_, adding the vertex *u*_1_; for *j* = 5 (*j* ≤ *k*), subdivide a second time, adding the vertex *u*_2_. Connect all new vertices to *w*_0_ to form a wheel. Call this graph $\bar{G_{j,k,5}}$ for 4 ≤ *j* ≤ *k* ≤ 5.

**Figure 23.**
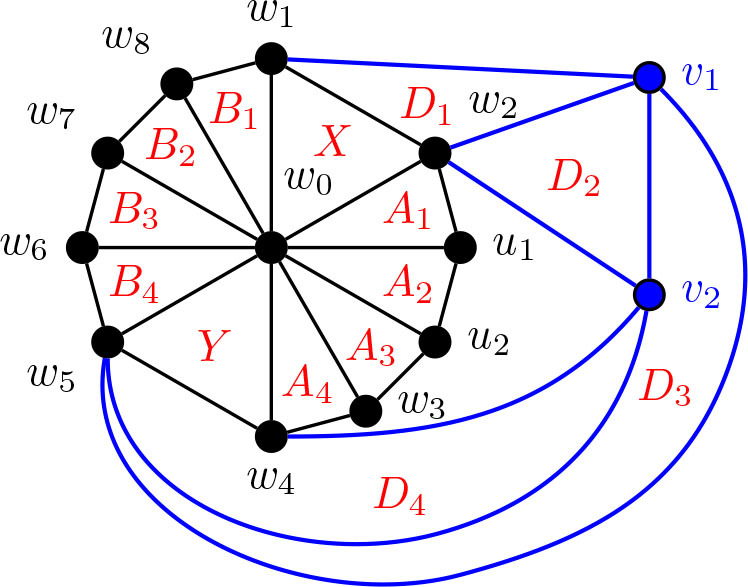
A graph $\bar{G_{5,5,5}}$ from Construction 5.28 such that *ℐ* (*G*_5,5,5_) = Θ 〈5, 5, 5〉

An example of the construction of $\bar{G_{5,5,5}}$ is given in Figure 23 below.

**Lemma 5.33.**

*If* $\bar{G_{j,k,5}}$ *is the graph constructed by Construction* 5.32, *then ℐ* (*G_j,k,_*_5_) = *𝒜* (*G_j,k,_*_5_) = Θ 〈*j, k,* 5〉 *for* 4 ≤ *j* ≤ *k* ≤ 5.

#### 5.4.2. Construction of *G̅* for Θ 〈*j*, *k*, *ℓ*〉 for 3 ≤ *j* ≤ *k* ≤ *ℓ*, *ℓ* ≥ 6

**Construction 5.34.**

Begin with a copy of the graph $\bar{G_{3,3,\ell }}$ from Construction 5.22 for Θ 〈3, 5, *ℓ*〉 for *ℓ* ≥ 6. For 3 ≤ *k* ≤ *ℓ*, subdivide the edge *w*_1_*w*_6_ *k −* 3 times, adding the new vertices *w*_7_, *w*_8_, . . . , *w_k_*_+3_. Join each of *w*_7_, *w*_8_, . . . , *w_k_*_+3_ to *w*_0_, so that *w*_0_, *w*_1_, . . . , *w_k_*_+3_ forms a wheel. Then, for 3 ≤ *j* ≤ *k*, subdivide the edge *w*_2_*w*_3_ *j −* 3 times, adding the new vertices *u*_1_, *u*_2_, . . . , *u_j−_*_3_. Again, join each of *u*_1_, *u*_2_, . . . , *u_j−_*_3_ to *w*_0_ to form a wheel. Call this graph $\bar{G_{j,k,\ell }}$ for 3 ≤ *j* ≤ *k* ≤ *ℓ* and *ℓ* ≥ 6.

**Lemma 5.35.**

*If* $\bar{G_{j,k,\ell }}$ *for* 3 ≤ *j* ≤ *k* ≤ *ℓ and ℓ* ≥ 6 *is the graph constructed by Construction* 5.34, *then ℐ* (*G*_*j*, *k*, *ℓ*_) = *𝒜* (*G*_*j*, *k*, *ℓ*_) = Θ 〈*j, k, ℓ*〉.

The lemmas above imply the sufficiency of Theorem 5.3: if a theta graph is not one of seven exceptions listed, then it is an *i*-graph. In the next section, we complete the proof by examining the exception cases.

**Figure 24.**
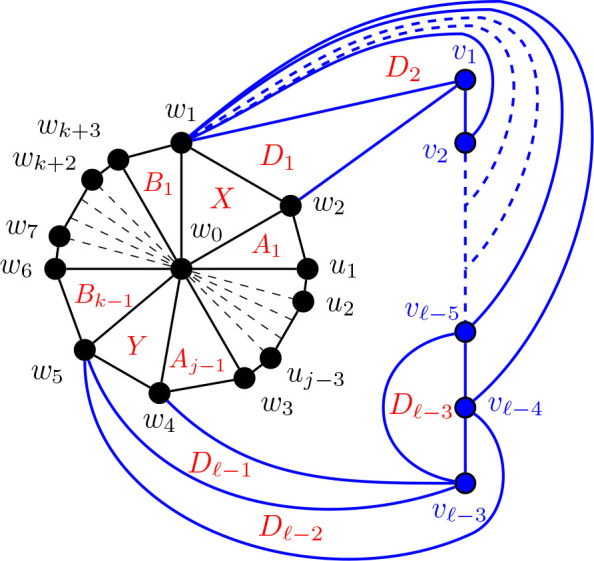
The graph $\bar{G_{j,k,\ell }}$ from Construction 5.34 such that *ℐ* (*G*_*j*, *k*, *ℓ*_) = Θ 〈*j, k, ℓ*〉 for 3 ≤ *j* ≤ *k* ≤ *ℓ* and *ℓ* ≥ 6

## 6. Theta graphs that are not *i*-graphs

In this section we show that Θ 〈2, 2, 4〉, Θ 〈2, 3, 3〉, Θ 〈2, 3, 4〉, and Θ 〈3, 3, 3〉 are not *i*-graphs; together with Proposition 3.7, this completes the proof of Theorem 5.3.

### 6.1. Θ 〈2, 2, 4〉 is not an *i*-graph

**Proposition 6.1.**

*The graph* Θ 〈2, 2, 4〉 *is not i-graph realizable.*

**Proof**

**Figure 25.**
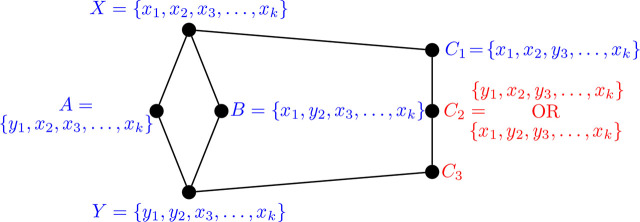
*H* = Θ 〈2, 2, 4〉 non-construction

Suppose to the contrary that Θ 〈2, 2, 4〉 is realizable as an *i*-graph, and that *H* = Θ 〈2, 2, 4〉 ≅ *ℐ* (*G*) for some graph *G*. Label the vertices of *H* as in Figure 25.

From Proposition 3.6, the composition of the following *i*-sets of *G* are immediate:

$$\begin{array}{rr} X=\left\{x_{1},x_{2},x_{3},\;\ldots ,x_{k}\right\}, & Y=\left\{y_{1},y_{2},x_{3},\;\ldots ,x_{k}\right\},\\ A=\left\{y_{1},x_{2},x_{3},\;\ldots ,x_{k}\right\}, & B=\left\{x_{1},y_{2},x_{3},\;\ldots ,x_{k}\right\},\\ C_{1}=\left\{x_{1},x_{2},y_{3},\;\ldots ,x_{k}\right\}, & \end{array}$$

where *k* ≥ 3 and *y*_1_, *y*_2_, *y*_3_ are three distinct vertices in *G − X*. These sets are illustrated in blue in Figure 25. This leaves only the composition of *C*_2_ and *C*_3_ (in red) to be determined. As we construct *C*_2_, notice first that *y*_3_ ∈ *C*_2_; otherwise, if say some other *z* ∈ *C*_2_ so that $C1\overset{y_{3}z}{∼}C_{2}$ , then $X\overset{x_{3}z}{∼}C_{2}$ , and *XC*_2_ ∈ *E*(*H*). Thus, a token on one of {*x*_1_, *x*_2_, *x*_4_, . . . , *x_k_*} moves in the transition from *C*_1_ to *C*_2_. We consider three cases.

Case 1: The token on *x*_1_ moves. If $C_{1}\overset{x_{1}z}{∼}C_{2}$ for some *z* ∉ {*y*_1_, *y*_2_}, then |*C*_2_ ∩ *Y*| = 3, contradicting the distance requirement between *i*-sets from Observation 3.2. Moreover, from the composition of *B*, *x*_1_ ≁ *y*_2_, and so $C_{1}\overset{x_{1}y_{1}}{∼}C_{2}$ , so that *C*_2_ = {*y*_1_, *x*_2_, *y*_3_, . . . , *x_k_*}. However, since *x*_3_ ∼ *y*_3_, we have that $A\overset{x_{3}y_{3}}{∼}C_{2}$ , so that *AC*_2_ ∈ *E*(*G*), a contradiction.

Case 2: The token on *x*_2_ moves. An argument similar to Case 1 constructs *C*_2_ = {*x*_1_, *y*_2_, *y*_3_, . . . , *x_k_*}, with $B\overset{x_{3}y_{3}}{∼}C_{2}$ , resulting in the contradiction *BC*_2_ ∈ *E*(*G*).

Case 3: The token on *x_i_* for some *i* ∈ {4, 5, . . . , *k*} moves. From the compositions of *X* and *Y*, *x_i_* is not adjacent to any of {*x*_3_, *y*_1_, *y*_2_}, so the token at *x_i_* moves to some other vertex, say *z*, so that $C_{1}\overset{x_{i}z}{∼}C_{2}$ and {*x*_1_, *x*_2_, *y*_3_, *z*} ⊆ *C*_2_. This again contradicts the distance requirement of Observation 3.2 as |*C*_2_ ∩ *Y*| = 4.

In all cases, we fail to construct a graph *G* with *ℐ* (*G*) ≅ Θ 〈2, 2, 4〉 and so conclude that no such graph exists.

### 6.2. Θ 〈2, 3, 3〉 is not an *i*-Graph

**Proposition 6.2.**

*The graph* Θ 〈2, 3, 3〉 *is not i-graph realizable.*

**Proof**

**Figure 26.**
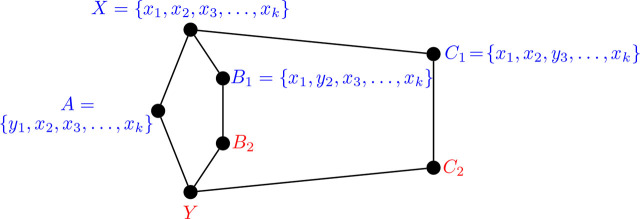
*H* = Θ 〈2, 3, 3〉 non-construction

To begin, we proceed similarly to the proof of Proposition 6.1: suppose to the contrary that Θ 〈2, 3, 3〉 is realizable as an *i*-graph, and that *H* = Θ 〈2, 3, 3〉 ≅ *ℐ* (*G*) for some graph *G*. Label the vertices of *H* as in Figure 26. As before, the corresponding *i*-sets in blue are established from previous results, and those in red are yet to be determined. Moreover, from the composition of these four blue *i*-sets, we observe that for each *i* ∈ {1, 2, 3}, *x_i_* ∼ *y_j_* if and only if *i* = *j*.

Unlike the construction for Θ 〈2, 2, 4〉, we no longer start with knowledge of the exact composition of *Y*. We proceed with a series of observations on the contents of the various *i*-sets:

(i) *y*_1_ ∈ *Y*, *y*_2_ ∈ *B*_2_, and *y*_3_ ∈ *C*_2_ by three applications of Proposition 3.6.
(ii) *y*_1_ ∉ *B*_2_ and *y*_1_ ∉ *C*_2_. If *y*_1_ ∈ *B*_2_, then $B_{1}\overset{x_{1}y_{1}}{∼}B_{2}$ (because *A* shows that *y*_1_ is not adjacent to *x*_3_, . . . , *x_k_*) so that *B*_2_ = {*y*_1_, *y*_2_, *x*_3_, . . . , *x_k_*}, and therefore $A\overset{x_{2}y_{2}}{∼}B_{2}$ , which is impossible. Similarly, if *y*_1_ ∈ *C*_2_, then $A\overset{x_{3}y_{3}}{∼}C_{2}$ , which is also impossible.
(iii) *y*_3_ ∉ *B*_2_. Otherwise, *B*_2_ = {*x*_1_, *y*_2_, *y*_3_, *x*_4_, . . . , *x_k_*} and so $C_{1}\overset{x_{2}y_{2}}{∼}B_{2}$ .
(iv) *y*_2_ ∉ *Y* and *y*_3_ ∉ *Y*. If *y*_2_ ∈ *Y*, then *Y* = {*y*_1_, *y*_2_, *x*_3_, . . . , *x_k_*} and so $B_{1}\overset{x_{1}y_{1}}{∼}Y$ . Likewise, if *y*_3_ ∈ *Y* then $C_{1}\overset{x_{1}y_{1}}{∼}Y$ .

From (i) and (ii), *y*_1_ ∈ *Y* but *y*_1_ ∉ *B*_2_, and similarly from (iv) *y*_2_ ∈ *B*_2_ but *y*_2_ ∉ *Y* ; therefore, $B_{2}\overset{y_{2}y_{1}}{∼}Y$ . Now, since *x*_1_ ∼ *y*_1_, and *y*_1_ ∈ *Y*, we have that *x*_1_ ∉ *Y*. Thus, if *x*_1_ were in *B*_2_, its token would move in the transition from *B*_2_ to *Y*. However, we have already established that it is the token at *y*_2_ that moves, and so *x*_1_ ∉ *B*_2_. We conclude that $B_{1}\overset{x_{1}z}{∼}B_{2}$ for some *z* ∉ {*y*_1_, *y*_3_}, so that *B*_2_ = {*z, y*_2_, *x*_3_, . . . , *x_k_*}. Notice that since *B*_2_ is independent, and *x*_2_ ∼ *y*_2_, it follows that *z* ≠ *x*_2_.

Using similar arguments, we determine that $C_{2}\overset{y_{3}y_{1}}{∼}Y$ , and that $C_{1}\overset{x_{1}w}{∼}C_{2}$ for some *w* ∉ {*y*_1_, *y*_2_, *x*_1_}. Moreover, *C*_2_ = {*w, x*_2_, *y*_3_, *x*_4_, . . . , *x_k_*}. Again, note that since *x*_3_ ∼ *y*_3_, *w* ≠ *x*_3_.

From $B_{2}\overset{y_{2}y_{1}}{∼}Y$ , we have that *Y* = {*y*_1_, *z, x*_3_, *x*_4_ . . . , *x_k_*}. However, from $C_{2}\overset{y_{3}y_{1}}{∼}Y$ , we also have that *Y* = {*y*_1_, *x*_2_, *w, x*_4_, . . . , *x_k_*}. As we have already established that *z* ≠ *x*_2_ and *w* ≠ *x*_3_, we arrive at two contradicting compositions of *Y*. Thus, no such graph *G* exists, and we conclude that Θ 〈2, 3, 3〉 is not an *i*-graph.

### 6.3. Θ 〈2, 3, 4〉 is not an *i*-Graph

**Proposition 6.3.**

*The graph* Θ 〈2, 3, 4〉 *is not i-graph realizable.*

**Proof**

**Figure 27.**
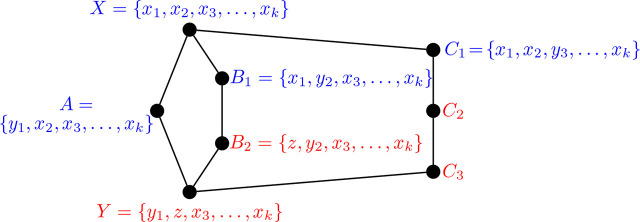
*H* = Θ 〈2, 3, 4〉 non-construction

The construction for our contradiction begins similarly to that of Θ 〈2, 3, 3〉 in Proposition 6.2. As before, we illustrate the graph in Figure 27, labelling the known sets in blue, and those yet to be determined in red. Given the similarity of Θ 〈2, 3, 3〉 and Θ 〈2, 3, 4〉, many of the observations from Proposition 6.2 carry through to our current proof. In particular, all of (i)–(iv) hold here, including that *y*_1_ ∉ *C*_2_ from (ii). Moreover, the compositions of *Y* and *B*_2_ also hold, where *z* is some vertex with *z* ∉ {*y*_1_, *x*_2_, *y*_2_}.

We now attempt to build *C*_2_. From Proposition 3.6, since *X* ≁ *C*_2_, *y*_3_ ∈ *C*_2_ (and *x*_3_ ∉ *C*_2_). From the distance requirement of Observation 3.2, |*X − C*_2_| ≤ 2, and so at least one of *x*_1_ or *x*_2_ is in *C*_2_. Recall from the construction for Proposition 6.2 that $A\overset{x_{2}z}{∼}Y$ and $B_{1}\overset{x_{1}z}{∼}B_{2}$ , and so *z* is adjacent to both *x*_1_ and *x*_2_. Hence, *z* ∉ *C*_2_.

Gathering these results shows that none of {*x*_3_, *z, y*_1_} are in *C*_2_, and thus, *d*(*C*_2_, *Y*) ≥ 3, contradicting the distance requirement of Observation 3.2. We conclude that no graph *G* exists such that *ℐ* (*G*) = Θ 〈2, 3, 4〉.

### 6.4. Θ 〈3, 3, 3〉 is not an *i*-Graph

**Proposition 6.4.**

*The graph* Θ 〈3, 3, 3〉 *is not i-graph realizable.*

**Proof**

**Figure 28.**
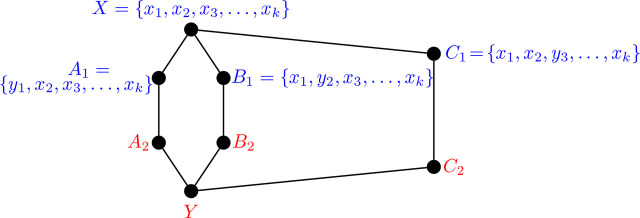
*H* = Θ 〈3, 3, 3〉 non-construction

Let *H* be the theta graph Θ 〈3, 3, 3〉, with vertices labelled as in Figure 28. Suppose to the contrary that there exists some graph *G* such that *H* is the *i*-graph of *G*; that is, *ℐ* (*G*) = Θ 〈3, 3, 3〉.

Since *d_H_*(*A*_1_, *Y*) = 2, by Observation 3.4, |*A*_1_ − *Y*| = 2. Similarly, |*B*_1_ − *Y*| = 2 and |*C*_1_*−Y*| = 2. Suppose that, say, *x*_4_ ∉ *Y*. Hence, by Observation 3.2 |{*x*_1_, *x*_2_, *x*_3_} ∩ *Y*| ≥ 1. Without loss of generality, say *x*_1_ ∈ *Y*. Then since *y*_1_ ∉ *Y* and *x*_4_ ∉ *Y*, both *x*_2_ and *x*_3_ ∈ *Y* to satisfy |*A*_1_ − *Y*| = 2. However, then *Y* = {*x*_1_, *x*_2_, *x*_3_, *z, x*_5_, . . . , *x_k_*} for some vertex *z* ∼ *x*_4_, and so $X\overset{x_{4}z}{∼}Y$ , which is not so. We therefore conclude that *x*_4_ ∈ *Y*, and likewise *x_i_* ∈ *Y* for *i* ≥ 4. Thus, {*x*_1_, *x*_2_, *x*_3_} ∩ *Y* = ∅.

Returning to *A*_1_, since *d*(*A*_1_, *Y*) = 2 and *x*_2_, *x*_3_ ∉ *Y*, we have that *y*_1_ ∈ *Y*. Similarly, *y*_2_, *y*_3_ ∈ *Y*. Thus, *Y* = {*y*_1_, *y*_2_, *y*_3_, *x*_3_, . . . , *x_k_*}. Moreover, *A*_2_ is obtained from *A*_1_ by replacing one of *x*_2_ or *x*_3_, by *y*_2_ or *y*_3_, respectively. Say, $A_{1}\overset{x_{2}y_{2}}{∼}A_{2}$ so that *A*_2_ = {*y*_1_, *y*_2_, *x*_3_, . . . , *x_k_*}. Now, however, we have that $B_{1}\overset{x_{1}y_{1}}{∼}A_{2}$ , but clearly *B*_1_ ≁ *A*_2_. It follows that Θ 〈3, 3, 3〉 is not an *i*-graph.

This completes the proof of Theorem 5.3.

## 7. Other results

In this section we first display a graph that is neither a theta graph nor an *i*-graph, and then use the method of graph complements to show that every cubic 3-connected bipartite planar graph is an *i*-graph.

### 7.1. A non-theta non-*i*-graph

So far, every non-*i*-graph we have observed is either one of the seven theta graphs from Theorem 5.3, or contains one of those seven as an induced subgraph (as per Corollary 3.11). This leads naturally to the question of whether theta graphs provide a forbidden subgraph characterization for *i*-graphs. Unfortunately, this is not the case.

**Figure 29.**
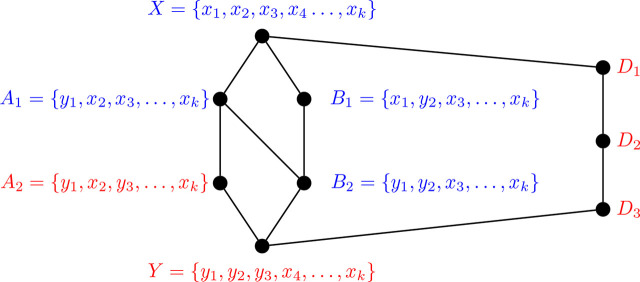
A non-theta non-*i*-graph 𝒯.

Consider the graph 𝒯 in Figure 29: it is not a theta graph, and although it contains several theta graphs as induced subgraphs, none of those induced subgraphs are among the seven non-*i*-graph theta graphs. In Proposition 7.1 we confirm that 𝒯 is not an *i*-graph.

**Proposition 7.1.**

*The graph* 𝒯 *in Figure* 29 *is not an i-graph.*

**Proof**

We proceed similarly to the proofs for the theta non-*i*-graphs in Section 6. Let 𝒯 be the graph in Figure 29, with vertices as labelled, and suppose to the contrary that there is some graph *G* such that *ℐ* (*G*) ≅ 𝒯.

To begin, we determine the vertices of the two induced *C*_4_’s of 𝒯. Immediately from Proposition 3.6, the vertices of *X*, *A*_1_, *B*_1_, and *B*_2_ are as labelled in Figure 29. Using a second application of Proposition 3.6, *A*_2_ differs from *A*_1_ in exactly one position, different from *B*_2_. Without loss of generality, say, $A_{1}\overset{x_{3}y_{3}}{∼}A_{2}$ . Then again by the proposition, *Y* = {*y*_1_, *y*_2_, *y*_3_, *x*_4_ . . . , *x_k_*} as in Figure 29.

We now construct the vertices of *D*_1_ through a series of claims:

(i) *x*_3_ is not in *D*_1_.
  From Lemma 3.3, *D*_1_ differs from *X* in one vertex, different from both *A*_1_ and *B*_1_; moreover, *x*_1_ and *x*_2_ are in *D*_1_. Notice that {*x*_4_, *x*_5_, . . . , *x_k_*} ⊆ *D*_1_: suppose to the contrary that, say, *x*_4_ ∉ *D*_1_, so that $X\overset{x_{4}z}{∼}D_{1}$ . Then *z* ≠ *y*_1_, since *y*_1_ ∼*_G_ x*_1_ and *D*_1_ is independent. Likewise *z* ≠ *y*_2_ and *z* ≠ *y*_3_. Thus *D*_1_ = {*x*_1_, *x*_2_, *x*_3_, *z, x*_5_, . . . , *x_k_*} has |*Y* ∩ *D*_1_| = 4, but *d*(*D*_1_, *Y*) = 3, contradicting Observation 3.4.
(ii) *y*_1_, *y*_2_, and *y*_3_ are not in *D*_1_.
  As above, *y*_1_ and *y*_2_ are not in *D*_1_, as *D*_1_ is an independent set containing *x*_1_ and *x*_2_. Suppose to the contrary that $X\overset{x_{3}y_{3}}{∼}D_{1}$ , so that *D*_1_ = {*x*_1_, *x*_2_, *y*_3_, . . . , *x_k_*}. Then, since *x*_1_ ∼*_G_ y*_1_, we have that $D_{1}\overset{x_{1}y_{1}}{∼}A_{2}$ , a contradiction.
(iii) *D*_1_ = {*x*_1_, *x*_2_, *z, x*_4_, . . . , *x_k_*}, where *z* ∉ {*y*_1_, *y*_2_, *y*_3_}.
  Immediate from (i) and (ii).

A contradiction for the existence of *G* arises as we construct *D*_2_. Since *d*_𝒯_(*D*_1_, *Y*) = 3 and |*D*_1_ ∩ *Y*| = 3, at each step along the path through *D*_2_, *D*_3_, and *Y*, exactly one token departs from a vertex of *D*_1_ and moves to one of *Y − D*_1_ = {*y*_1_, *y*_2_, *y*_3_}. However, *D*_2_ ≠ {*y*_1_, *x*_2_, *z, x*_4_, . . . , *x_k_*}, since otherwise, $A_{1}\overset{x_{3}z}{∼}D_{2}$ . Likewise, *D*_2_ ≠ {*x*_1_, *y*_2_, *z, x*_4_, . . . , *x_k_*}. Finally *D*_2_ ≠ {*x*_1_, *x*_2_, *y*_3_, *x*_4_, . . . , *x_k_*} as again we have $A_{2}\overset{y_{1}x_{1}}{∼}D_{2}$ . Thus, we cannot build a set *D*_2_ such that |*D*_2_ ∩ *Y*| ≤ 2 as required. We so conclude that no such graph *G* exists, and that 𝒯 is not an *i*-graph.

Like the seven non-*i*-graph realizable theta graphs of Theorem 5.3, 𝒯 is a minimal obstruction to a graph being an *i*-graph; every induced subgraph of 𝒯 is a theta graph.

### 7.2. Maximal planar graphs

We conclude this section with a result to demonstrate that certain planar graphs are *i*-graphs and *α*-graphs. Our proof uses the following three known results.

**Theorem 7.2 ([3, Theorem 4.6]).**

*A cubic graph is* 3-*connected if and only if it is* 3-*edge connected.*

**Theorem 7.3 ([11]).**

*A connected planar graph G is bipartite if and only if its dual G̃ is Eulerian.*

**Theorem 7.4 ([6, 10]).**

*A maximal planar graph G of order at least* 3 *has χ*(*G*) = 3 *if and only if G is Eulerian.*

In our proof of the following theorem, we consider a graph *G* that is cubic, 3-connected, bipartite, and planar. We then examine its dual *G̃*, and how those specific properties of *G* translate to *G̃*. Then, we construct the complement of *G̃*, which we refer to as *H*. We claim that *H* is a seed graph of *G*; that is, *ℐ* (*H*) contains an induced copy of *G*.

**Theorem 7.5.**

*Every cubic* 3-*connected bipartite planar graph is an i-graph and an *α*-graph.*

**Proof**

Let *G* be a cubic 3-connected bipartite planar graph and consider the dual *G̃* of *G*. Since *G* is bridgeless, *G̃* has no loops. Moreover, since *G* is 3-connected, Theorem 7.2 implies that no two edges separate *G*; hence, *G̃* has no multiple edges. Therefore, *G̃* is a (simple) graph. Further, since *G* is cubic, each face of *G̃* is a triangle, and so *G̃* is a maximal planar graph.

We note the following two key observations. First, since each face of *G̃* is a triangle,

(1) each edge of *G̃* belongs to a triangle.
  For the second, note that since *G* is bipartite, *G̃* is Eulerian (by Theorem 7.3). Then, by Theorem 7.4, *χ*(*G̃*) = 3, so *G̃* does not contain a copy of *K*_4_. Thus,
(2) every triangle of *G̃* is a maximal clique.
  Now, by the duality of *G̃* and *G*, there is a one-to-one correspondence between the facial triangles of *G̃* and the vertices of *G*. Let *H* be the complement of *G̃*. By (1) and (2), *i*(*H*) = *α*(*H*) = 3, and every maximal independent set of *G* corresponds to a triangle of *G̃*. But again, by duality, the facial triangles of *G̃* and their adjacencies correspond to the vertices of *G* and their adjacencies. Therefore, *ℐ* (*H*) contains *G* as an induced subgraph. Any additional unwanted vertices of *ℐ* (*H*) can be removed by applying the Deletion Lemma (Lemma 3.10).

## 8. Open problems

We conclude with a few open problems. Although the problems are stated here for *i*-graphs, many are relevant to other reconfiguration graphs pertaining to domination-type parameters and are also mentioned in [8, 9].

**Problem 2.**

Determine conditions on the graph *G* under which *ℐ* (*G*) is (a) connected (b) disconnected.

A graph *G* is *well-covered* if all its maximal independent sets have the same cardinality, that is, if *α*(*G*) = *i*(*G*).

**Problem 3.**

(a) Determine those *i*-graphs that are also *α*-graph realizable.
(b) Determine those *i*-graphs *H* for which there exists a well-covered seed graph *G* such that *ℐ* (*G*) ≅ *H*.

**Problem 4.**

We have already seen that classes of graphs such as trees, cycles, and, more generally, block graphs, are *i*-graphs.

As we build new graphs from these families of *i*-graphs, using tree structures, which of those are also *i*-graphs? For example, are cycle-trees *i*-graphs? Path-trees? For which families of graphs *H* are *H*-trees *i*-graphs?

**Problem 5.**

Determine the structure of *i*-graphs of various families of trees. For example, consider

(a) caterpillars in which every vertex has degree 1 or 3,
(b) spiders (*K*_1,*r*_ with each edge subdivided).

**Problem 6.**

Find more classes of *i*-graphs that are Hamiltonian, or Hamiltonian traceable.

**Problem 7.**

Suppose *G*_1_, *G*_2_, . . . are graphs such that *ℐ* (*G*_1_) ≅ *G*_2_, *ℐ* (*G*_2_) ≅ *G*_3_, *ℐ* (*G*_3_) ≅ *G*_4_, . . . . Under which conditions does there exist an integer *k* such that *ℐ* (*G_k_*) ≅ *G*_1_?

As a special case of Problem 7, note that for any *n* ≥ 1, *ℐ* (*K_n_*) ≅ *K_n_*, and that for *k* ≡ 2 (mod 3), *ℐ* (*C_k_*) ≅ *C_k_*.

**Problem 8.**

Characterize the graphs *G* for which *ℐ* (*G*) ≅ *G*.
